# Human endothelial colony forming cells (ECFCs) require endothelial protein C receptor (EPCR) for cell cycle progression and angiogenic activity

**DOI:** 10.1007/s10456-025-09982-8

**Published:** 2025-05-23

**Authors:** Sarah E. J. Chambers, Jasenka Guduric-Fuchs, Edoardo Pedrini, Pietro M. Bertelli, Chutima Charoensuk, Elisa Peixoto, Varun Pathak, Hamza I. Alhamdan, Ruoxiao Xie, Anna Krasnodembskaya, Judith Lechner, Alan W. Stitt, Reinhold J. Medina

**Affiliations:** 1 Wellcome-Wolfson Institute for Experimental Medicine, School of Medicine, Dentistry, and Biomedical Science, Queen’s University Belfast, Belfast, UK; 2 Center for Omics Sciences, Vita-Salute San Raffaele University, 20132 Milan, Italy; 3 Faculty of Medicine, Ibn Sina University for Medical Sciences, Amman, Jordan; 4 Department of Eye and Vision Science, Institute of Life Course and Medical Sciences, Faculty of Health and Life Sciences, University of Liverpool, Liverpool, UK; 5 Department of Materials, Design and Manufacturing Engineering, School of Engineering, University of Liverpool, Liverpool, UK

**Keywords:** Protein C receptor, Endothelial progenitor, Endothelial colony forming cells, Cell cycle, Angiogenesis, Transforming growth factor beta

## Abstract

Vascular repair and regeneration are critical for tissue homeostasis. Endothelial colony forming cells (ECFCs) are vessel-resident progenitors with vasoreparative capacity and they offer an important avenue for allogeneic cytotherapy to achieve perfusion of ischemic tissues. Endothelial Protein C Receptor (EPCR) has been proposed as a marker for vascular endothelial stem cells, but its precise role in ECFC biology remains unknown. The current study has investigated the biological relevance of EPCR in ECFC function. Our data show that over 95% of ECFCs exhibit high EPCR expression. These levels surpassing CD34 and CD157, positions EPCR as a new robust ECFC immunophenotypic marker, alongside established markers CD31 and CD105. Functionally, depleting EPCR expression in ECFCs significantly diminished angiogenic activity, including proliferation, migration and tube formation. This knockdown also altered normal ECFC barrier function. Transcriptomic analysis indicated that knockdown of EPCR led to enrichment of gene signatures for cell cycle, TGF beta, and focal adhesion kinases. G1 cell cycle arrest was confirmed in ECFCs with depleted EPCR. Mechanistically, EPCR knockdown led to increased release of TGFβ2 and SMAD2/3 activation, coupled with increased p21, decreased pFAK, and increased transgelin. Additionally, we showed that quiescent ECFCs showed significantly lower EPCR expression when compared to proliferating ECFCs. In agreement with this, cell sorting experiments demonstrated that ECFCs with the highest EPCR expression exhibited the highest clonogenic capacity. In summary, our findings highlight that EPCR expression in ECFCs is critical for their angiogenic activity, by modulating cell cycle progression.

**Graphical abstract:**

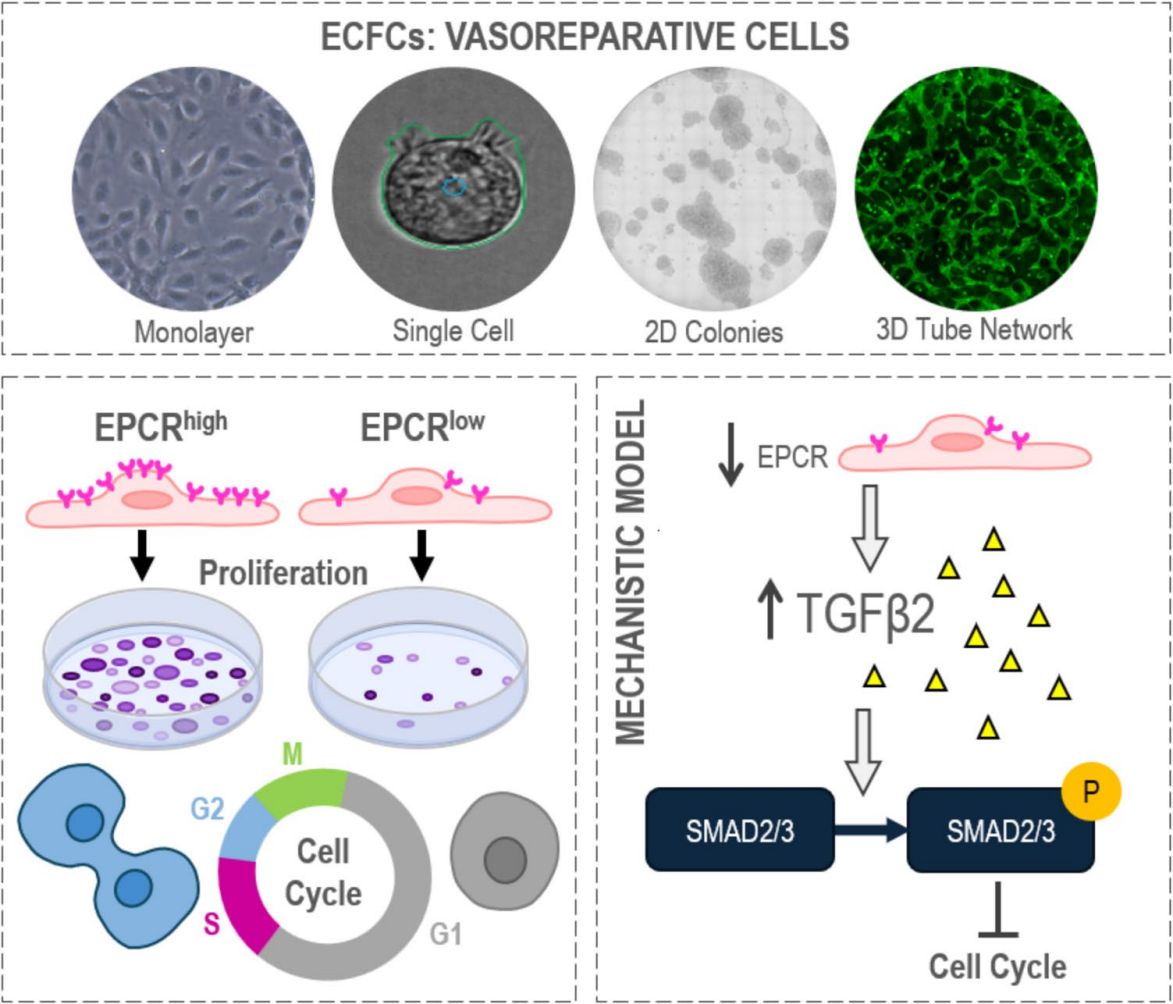

**Supplementary Information:**

The online version contains supplementary material available at 10.1007/s10456-025-09982-8.

## Introduction

Umbilical cord blood-derived endothelial colony forming cells (ECFCs) have the capacity for self-renewal and formation of de novo vasculature in vivo [1]. As such, ECFCs carry potential as a cell therapy for ischemic tissues [2] and have shown efficacy in an array of preclinical models of vascular insufficiency [3]. Importantly, an international survey provided technical guidance on how to standardize ECFC isolation [4]. ECFCs are commonly distinguished from other endothelial cell-types by their superior clonogenic and angiogenic capacities, highly pure immunophenotyping profiles based on CD31, VE-Cadherin, CD105, and VEGFR2 endothelial markers while lacking hematopoietic markers CD45 and CD14 [5]. ECFCs progenitor cell properties have been attributed to the expression of CD34 [6, 7], but more recently, CD157 [8] and *PROCR* (EPCR) [9] have been highlighted in vivo as identifiers of distinct populations of vascular endothelial stem cells. Isolation of ECFCs in vitro based on these markers, yields colonies of high proliferative potential that are capable of long term-self-renewal. Added to this, a cohort of human cord blood circulating ECFCs of CD34^bright^ cells possessed high clonal proliferative potential, compared to CD34^high^ cells, and were enriched in expression of *PROCR* (EPCR) [10].

Other than being used as a marker of stemness, it is well understood that EPCR enhances the activation of protein C by the thrombin-thrombomodulin complex into APC (activated protein C) [11]. EPCR is also required as a co-receptor for noncanonical proteolytic cleavage of PAR-1 (at Arg46) to elicit cytoprotective effects of APC, and indeed capable of switching thrombin-mediated Arg41 cleavage of PAR-1 from pro-inflammatory to anti-inflammatory by recruiting β-Arrestins to the plasma membrane [12]. This EPCR-PAR-1 biased signaling has recently been described to be crucial in restoration of blood flow in post-ischemic reperfusion in vivo [13].

The aim of this study is to investigate the functional role of EPCR in human umbilical cord blood-derived ECFCs using established protocols of endothelial potency (colony formation, tubulogenesis, migration, and endothelial barrier formation). Here we uncovered a key role for EPCR as a regulator of cell cycle progression in ECFCs, via its downstream TGFβ2 signaling. We demonstrate by selection of ECFCs with low EPCR expression, either by transient knockdown or by fluorescence activated cell sorting, that ECFCs highly proliferative state is dependent on the expression of EPCR.

## Results

### EPCR is highly expressed in the cellular membrane of human cord blood-derived ECFCs and during mouse lung microvascular regeneration

**Fig. 1:**
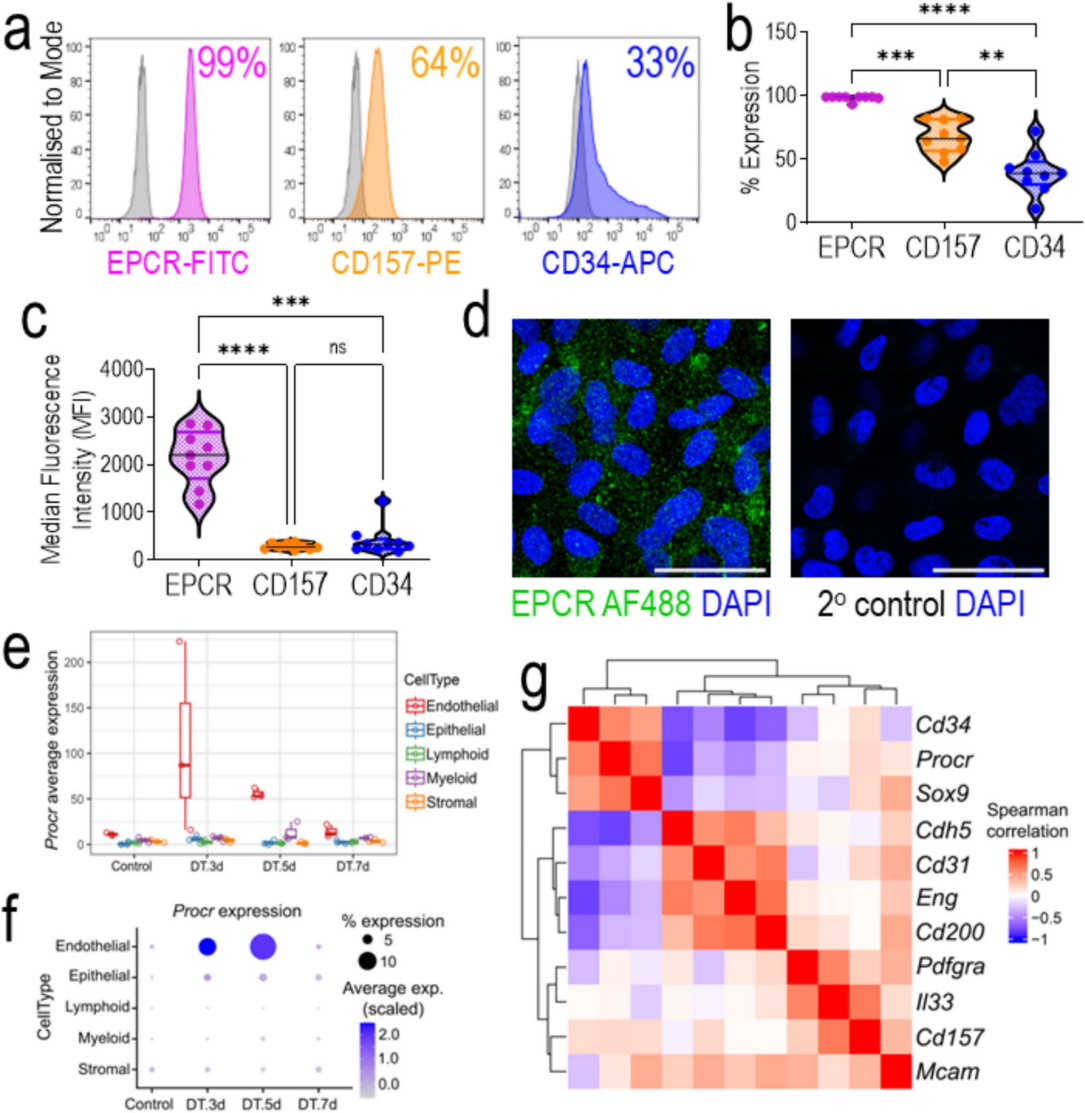
ECFCs show widespread expression of EPCR on cell surface. **a** Cell surface expression by flow cytometry of EPCR (pink histogram), CD157 (orange histogram), and CD34 (blue histogram) relative to unstained sample (grey histogram). Violin plots show distribution of (**b**) % Expression and (**c**) MFI (median fluorescence intensity) for EPCR, CD157, and CD34, n = 9. **d** Immunofluorescence of EPCR (AF488) in green shows widespread distribution on cell surface. Nucleus stained with DAPI in blue. Scale bar 50 µm. One-way ANOVA was performed, ns = not significant, **p < 0.01; ***p < 0.001, ****p < 0.0001. **e** *Procr* average expression by cell type from a model of severe acute lung injury GSE211335. **f** *Procr* percentage expression across lung cell types and timepoints after injury **g** Spearman correlation of *Procr* transcript with reported markers of endothelial stemness and differentiation

ECFCs can be identified by a combination of cell surface markers [5]. Previously we have demonstrated that ECFCs exhibit high expression of endothelial markers CD31 and CD105, low/negligible expression of hematopoietic markers CD14 and CD45, and varied expression of stem/progenitor marker CD34 [2]. Since EPCR has been identified as a marker of vascular endothelial stem cells in mice [9], we assessed its expression in human cord blood-derived ECFCs. Here we show cell surface expression of EPCR, CD157, and CD34 on ECFCs (Fig. 1a). The % of expression and the median fluorescence intensity are both significantly higher for EPCR when compared to CD157 and CD34 (Fig. 1b, c). EPCR expression was also the most homogeneous among ECFCs from nine different donors as demonstrated by all assessments showing > 95% positivity. The expression of CD157 and CD34 was heterogeneous. CD157 exhibited expression levels from 48 to 82%, and CD34 from 11 to 72% (Fig. 1b). In addition, immunofluorescence of EPCR confirmed its widespread expression on the cell surface (Fig. 1d). To corroborate our findings, we assessed ECFC transcriptomes generated by other groups [14]. Publicly available data from GEO with IDs GSE263058 and GSE131995 were analyzed [15, 16]. We found that human cord blood-derived ECFCs showed significant higher *PROCR* mRNA expression than human induced pluripotent stem cells (Supplementary Fig. 1a). Similarly, peripheral blood-derived ECFCs exhibited a significantly higher expression of *PROCR* mRNA than subcutaneous adipose tissue-derived stromal vascular fraction (Supplementary Fig. 1b). Furthermore, to establish the biological relevance of EPCR in the in vivo setting, we harnessed data from the single-cell transcriptomic atlas from a mouse model of lung microvascular regeneration [17], which is publicly available in GEO with ID GSE211335. This model of severe acute lung injury is induced by intratracheal instillation of diphtheria toxin in mice genetically modified to express the human diphtheria receptor in endothelial cells. This leads to the selective ablation of more than 70% of lung endothelial cells, which triggers vasoreparative mechanisms that achieve replenishment of endothelial cells by 7 days post-injury. Focusing on the expression of *Procr*, the gene that codes for EPCR, in the different cell types within this acute lung injury model, we confirmed that *Procr* was expressed at higher levels in mouse lung endothelial cells in comparison to other lung cell types (Fig. 1e), and there was a clear upregulation in *Procr* expression in response to injury. The increase in *Procr* expression was specific to endothelial cells and was quantified to be over two-fold increase by day 3 in 9.68% of cells (Fig. 1f). A gene correlation analysis of *Procr* with other reported marker genes for endothelial progenitor/stem cells, identified *Procr* clustering together with *Cd34* and *Sox9* having a positive strong correlation (Fig. 1g). Interestingly, there was a negative correlation between *Procr* and endothelial differentiation markers including *Cdh5* (Ve-cadherin), *Cd31* (Pecam), and *Eng* (Endoglin). Moreover, a second in vivo mouse lung injury model was examined. This model employed LPS to induce lung endothelial injury through inflammation and trigger vasoreparative processes [18]. Results indicated that *Procr* increased significantly after LPS exposure (Supplementary Fig. 2a). This *Procr* mRNA expression increase was already evident 6 h post LPS, reached highest levels at 24 h post LPS, and was not detectable at 72 and 168 h post LPS (Supplementary Fig. 2b). This suggested that EPCR expression in endothelial cells was associated with acute endothelial injury and early reparative mechanisms. Taken altogether, both the murine lung and human ECFC results confirm that EPCR (*PROCR*) is a robust marker for vasoreparative cells.

### In vitro cell functionality in ECFCs is dependent on EPCR presence

**Fig. 2:**
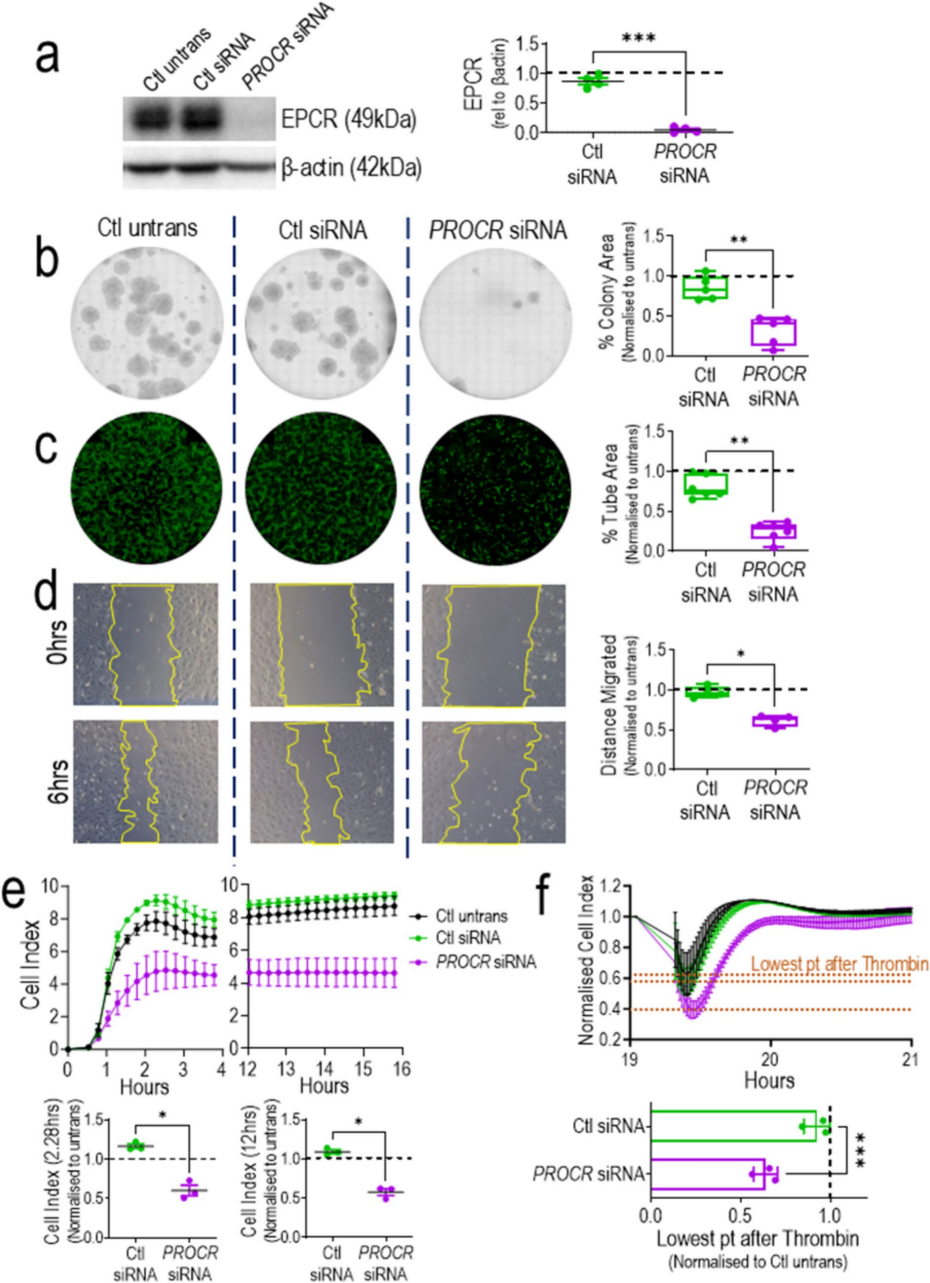
Expression of EPCR in ECFCs is essential for endothelial functionality. **a** *PROCR* silencing by *PROCR* siRNA confirmed in Western blot. Quantification of protein levels showed significant reduction in EPCR expression (49 kDa) relative to β-actin (42 kDa) in *PROCR* siRNA group compared to control untransfected and control siRNA groups, n = 4. Endothelial cell functional assays performed comparing control untransfected, control siRNA, and *PROCR* siRNA groups to include: **b** clonogenics assay and quantification of % colony area, n = 5, **c** tubulogenesis assay and quantification of % tube area, n = 6, **d** scratch migration assay from 0 to 6 h and quantification of distance migrated, n = 4, **e** barrier formation and quantification of cell index at 2.28 h and 12 h, n = 3 and **f** normalized cell index after thrombin treatment and quantification, n = 3. In all assays, control siRNA and *PROCR* siRNA groups are normalized to control untransfected, which is represented by the black dashed line, and analyzed by paired t-test. *p < 0.05, **p < 0.01; ***p < 0.001

To determine the role of EPCR expression on ECFCs functionality, *PROCR*, the gene coding for EPCR, was knocked down by siRNA. Protein evaluation by Western blot indicated a 95% decrease in EPCR expression (Fig. 2a, Supplementary Fig. 3). This knockdown led to a significant decrease in proliferative, tubulogenic, and migratory capacities, as shown by clonogenics (Fig. 2b), tubulogenesis (Fig. 2c), and scratch migration assays (Fig. 2d). In addition, endothelial barrier formation (Fig. 2e) is impeded when *PROCR* is silenced in ECFCs, measured by a significant reduction in cell index recorded during barrier formation (2.28 h) and after plateau (12 h). The full trace of barrier formation and stability over 18 h is available in Supplementary Fig. 4. Disruption of the endothelial barrier with thrombin treatment highlighted a greater response in *PROCR* siRNA cells compared to control siRNA, with a significant reduction in normalized cell index and a delay in barrier recovery after thrombin (Fig. 2f). In addition, we also evaluated the role of EPCR in more complex in vitro models including the 3D vasculature-on-chip and the MSC aggregates model. In agreement with previous results, EPCR^high^ ECFCs showed a significant larger area for sprouting and vascular network formation than EPCR^low^ ECFCs (Supplementary Fig. 5). Human MSC aggregates were cocultured with EPCR knocked down ECFCs or controls. We observed less intra-aggregate RFP vascular structures in EPCR knocked down ECFCs than in control ECFCs (Supplementary Fig. 6). Taken together, our evidence demonstrated that ECFCs in vitro functionality is dependent on the presence of EPCR.

### ECFCs with knocked-down *PROCR* exhibited a negative enrichment for cell cycle gene signature

**Fig. 3:**
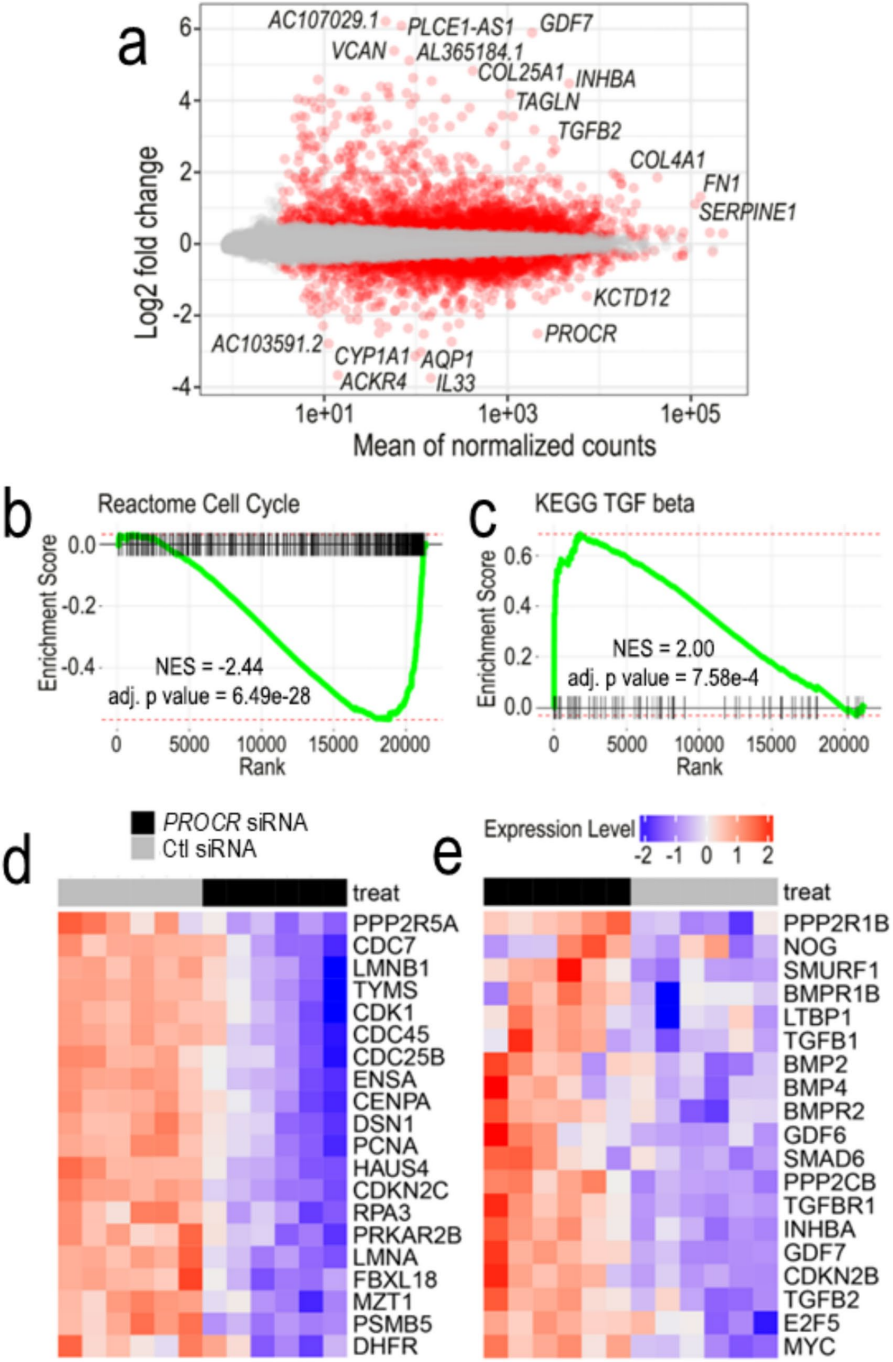
ECFCs with knocked-down EPCR by *PROCR* siRNA exhibited a negative enrichment for cell cycle gene signature. Bulk RNA-seq of 6 biological replicates highlighted 1,101 differentially expressed genes presented in (**a**) MA plot of *PROCR* siRNA vs control siRNA, n = 6. KEGG pathway analysis revealed that among the most down regulated gene signatures was CELL_CYCLE (**b**), and among the most up regulated was TGF_BETA_SIGNALING_PATHWAY (**c**). Heatmap showing cell cycle associated genes significantly downregulated (**d**) and upregulated (**e**) in *PROCR* siRNA compared to control siRNA

Having observed significant functional decline in ECFCs with *PROCR* silencing, bulk RNA sequencing was performed with six biological replicates of ECFCs transfected with *PROCR* siRNA vs control siRNA. We found 1,101 differentially expressed genes, 727 upregulated and 374 down regulated, as shown in the MA plot (Fig. 3a). KEGG pathway analysis revealed that the most down regulated gene signatures were DNA_REPLICATION and CELL_CYCLE, and the most up regulated gene signatures were FOCAL_ADHESION and TGF_BETA_SIGNALING_PATHWAY (Supplementary Fig. 7). Gene Set Enrichment Analysis (GSEA) for the REACTOME cell cycle gene signature confirmed a significant negative enrichment (Fig. 3b, d). We also found, as expected, that the TGF beta pathway gene signature was significantly enriched (Fig. 3c, e). Another link of *Procr* expression with cell proliferation was observed in the single cell RNA-seq atlas from mouse lung injury. Results from this mouse dataset indicated that *Procr* expression was the highest in proliferative lung capillaries, as annotated by the authors, in 50% of cells, at day 3 after injury (Supplementary Fig. 8a). Further gene correlation analysis identified *Procr* expression to be positively correlated with Apelin (*Apln*), Ki67 (*Mki67*), *Nrarp*, *Hmgb1*, and *Col18a1* (Supplementary Fig. 8b).

### Knocking down PROCR in ECFCs induced cell cycle arrest in G1

**Fig. 4:**
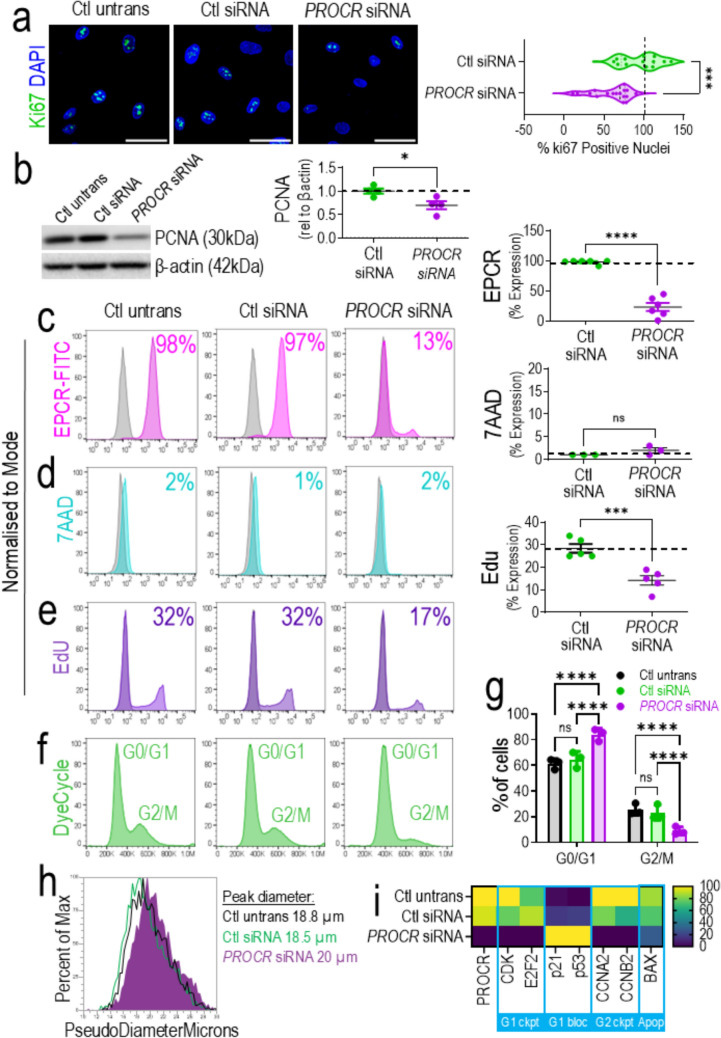
Knocking down *PROCR* in ECFCs halted the cell cycle in G1 phase. **a** Immunofluorescence staining of Ki67 (AF488 in green) in *PROCR* siRNA group vs control untransfected and control siRNA, nucleus stained with DAPI (blue), scale bar 50 µm. Violin plots show % ki67 positive nuclei quantified, n = 3 biological replicates, each technical replicate has been plotted. **b** *PROCR* silencing leads to decrease in PCNA (30 kDa) expression in western blot, relative to β-actin (42 kDa); quantification shows significant decrease in PCNA expression in *PROCR* siRNA group compared to control untransfected and control siRNA groups, n = 4. Control untransfected, control siRNA, and *PROCR* siRNA groups were assessed by flow cytometry and % expression quantified (in all plots grey histogram represents unstained control). **c** EPCR expression (pink histogram) and quantification, n = 6, **d** 7AAD expression (blue histogram) and quantification, n = 3. **e** EdU cell proliferation assay (purple histogram) and quantification, n = 5. **f** Cell cycle analysis using Vybrant dye cycle by flow cytometry (green histogram) shows distribution of G0/G1 (1st peak) and G2/M (2nd peak) phases of cell cycle. **g** Quantification of % cells within G0/G1 and G2/M phases n = 4, analyzed by 2-way ANOVA. **h** Histogram of PseudoDiameterMicrons generated from automated image analysis (AIA) using Attune CytPix. **i** RT-qPCR displayed as heatmap shows changes in gene expression in *PROCR*, G1 checkpoint genes (CDK, E2F2), G1 blocker genes (p53, p21), G2/M checkpoint genes (CCNA2, CCNB2), and apoptosis related BAX gene, comparing control untransfected, control siRNA, and PROCR siRNA groups, n = 6. ns = not significant, *p < 0.05, **p < 0.01; ***p < 0.001, ****p < 0.0001. In all graphs, the dotted line represents the average of control untransfected group

Next, we performed an in-depth functional study of the cell cycle in *PROCR* knocked down ECFCs. First, established markers of proliferation Ki67 and PCNA were used to confirm that EPCR expression on ECFCs drives proliferation. In the *PROCR* siRNA group, Ki67 (Fig. 4a) and PCNA (Fig. 4b, Supplementary Fig. 9) were significantly reduced compared to the control siRNA group. Secondly, cell surface immunophenotyping by flow cytometry, showed that the cell cycle arrest was not associated with increased cell death. ECFCs did not undergo apoptosis and remained viable, as shown by the lack of uptake of 7AAD, when EPCR was knocked down by ~ 84% (Fig. 4c, d). We found reduced incorporation of EdU, to around half when *PROCR* is silenced (Fig. 4e) and a reduction of G2/M peak using DyeCycle stain (Fig. 4f), which indicated an arrest in the G1 phase of the cell cycle. When quantified, there is a significant increase in cells within G1, and a concomitant reduction in cells within G2/M phases (Fig. 4g). Furthermore, we used the flow cytometry-based CytPix technology and automated image analysis (AIA) to define cell cycle status, which further confirmed the loss of cells in G2/M (Supplementary Fig. 11a-e). Beyond this, it showed an increase in the pseudo diameter of *PROCR* knocked down cells (Fig. 4h; Supplementary Fig. 11f). This correlates to morphological changes observed, where *PROCR* knocked down ECFCs appear bigger, more elongated, and lose their cobblestone morphology, compared to untransfected and control siRNA groups (Supplementary Fig. 12). This blockage of cell cycle progression is further confirmed by RT-qPCR gene expression data (Fig. 4h, Supplementary Fig. 10) which shows a significant reduction in expression of G1 checkpoint (CDK, E2F2) and G2/M checkpoint (CCNA2, CCNB2) genes, and a significant increase in expression of G1 blocker genes (p53, p21) when *PROCR* is knocked down. No significant change in apoptosis-related BAX gene agrees with previous 7AAD cell viability data.

### Diminished EPCR expression led to increased pSMAD2/3 and pERK

**Fig. 5:**
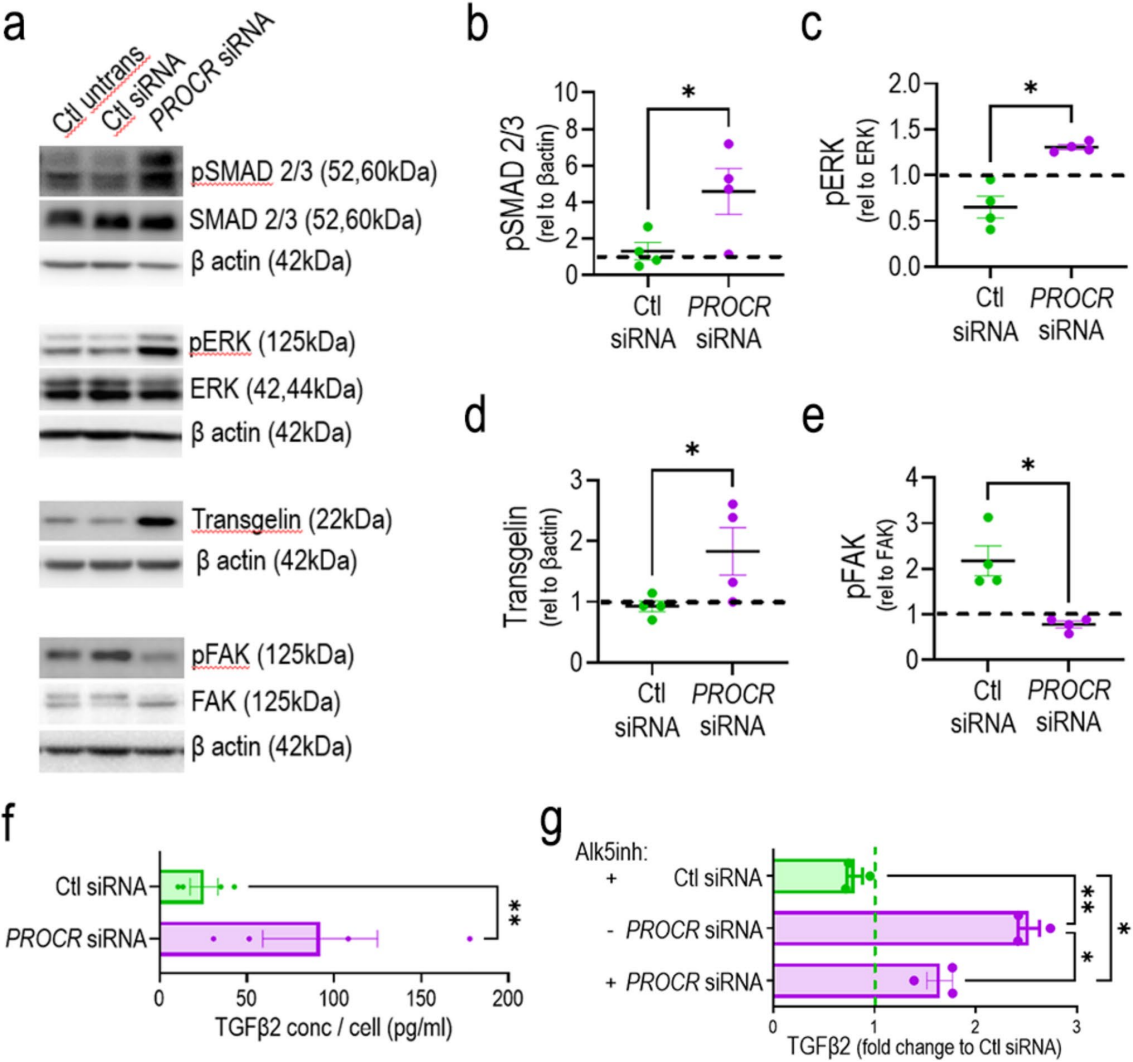
Diminished EPCR expression led to increased pSMAD2/3 and pERK via TGFβ2 signaling pathway as shown by Western blot (**a**) and densitometry quantification for pSMAD2/3 (**b**), pERK (**c**), Transgelin (**d**), and pFAK (**e**). **f** TGFβ2 secretion from control siRNA and *PROCR* siRNA assessed by ELISA. **g** Evaluation of TGFβ2 release when *PROCR* siRNA ECFCs were treated with Alk5 inhibitor SB525334. All data analyzed using a ratio paired t test, n = 4, *p < 0.05, **p < 0.01

To characterize the signaling pathways associated with the cell cycle arrest induced by the *PROCR* knockdown, we evaluated SMADs phosphorylation. Results highlight a significant increase in pSMAD2/3 (Fig. 5a, b) and no changes in pSMAD1/5 (Supplementary Fig. 13). In addition, we found a significant increase in pERK (Fig. 5a, c) and Transgelin (Fig. 5a, d), while there was a significant decrease in pFAK (Fig. 5a, e). Interestingly, the expression of Alk5, which is the receptor upstream of pSMAD2/3, showed a second band of lower molecular weight, evident only in *PROCR* knockdown ECFCs (Supplementary Fig. 13). Considering that Alk5 is activated by multiple ligands, we queried our transcriptome results for Alk5 ligand changes with *PROCR* knockdown. We evaluated eight Alk5 ligands: *TGFB1, TGFB2, TGFB3, INHBA, INHBB, GDF9, GDF11,* and *MSTN1* (Supplementary Fig. 14) and found a significant increase in the mRNA expression for *TGFB2, INHBA,* and *GDF11* when *PROCR* was knocked down. Thus, we measured TGFβ2 protein in the cell supernatant. ECFCs knocked down for *PROCR* showed a significant increase in TGFβ2 secretion in collected conditioned media, compared to control siRNA, with an average fold increase of 3.6 (Fig. 5f). We also tested the dependence of the TGFβ2 increase on SMAD2/3 signaling by employing the Alk5 inhibitor SB525334. We observed a significant decrease in TGFβ2 release in *PROCR* knocked down cells when treated with Alk5 inhibitor (Fig. 5g). Taken together, these data enabled us to propose a mechanistic model whereby the reduced expression of EPCR induced TGFβ2 release, which acts in an autocrine manner by binding to Alk5 and promoting phosphorylation of SMAD2/3, leading to increased pERK and Transgelin, with decreased pFAK.

### EPCR levels are decreased in quiescent ECFCs

**Fig. 6:**
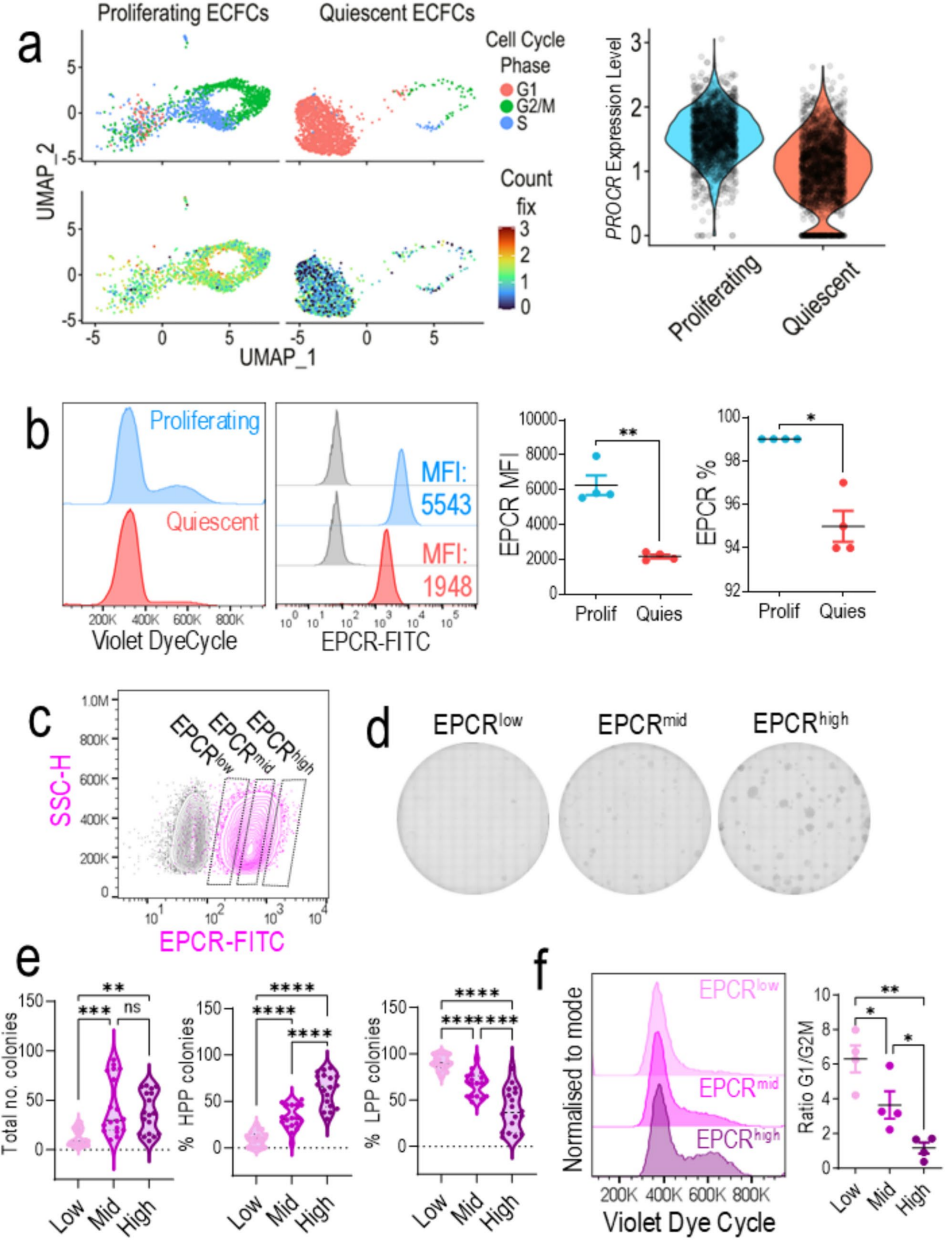
*PROCR* mRNA expression levels are associated with proliferative status in ECFCs **a** Expression of *PROCR* in scRNAseq of quiescent vs proliferating ECFCs. *PROCR* expression sub-divided by cell cycle phases using gene signatures, G1 (blue), G2M (yellow), S (red). **b** Cell cycle distribution by flow cytometry confirms quiescent ECFC monolayers (red histogram) are arrested in G1 phase and have loss of G2M peak compared to proliferating ECFCs (blue histogram). EPCR expression is presented as median fluorescence intensity (MFI). Grey histograms represent unstained control. Quantification of EPCR MFI and % expression analyzed by paired t-test, n = 4, *p < 0.05, **p < 0.01. **c** Contour plot shows gating strategy for EPCR^low^, EPCR^mid^, and EPCR^high^ populations which were sorted (by FACS) and plated for **d** clonogenic assay. **e** Violin plots show quantification of total number of colonies, as well as % high proliferative potential (HPP) and % low proliferative potential (LPP). Data analyzed by one-way ANOVA, ns = not significant, **p < 0.01, *** p < 0.001, ****p < 0.0001, n = 3 biological replicates, each technical replicate has been plotted. **f** Sorted populations of EPCR^low^, EPCR^mid^, and EPCR.^high^ were analyzed by flow cytometry for cell cycle distribution using Violet dye cycle and the ratio of G1/G2M populations quantified using one-way ANOVA, n = 4, *p < 0.05, **p < 0.01

To validate our results in a different model without gene knockdown, we use the quiescence cell model. ECFCs were cultured at high density until fully confluent, kept for 3 days and then serum starved, before sampling. Single cell RNA sequencing of ECFCs, made quiescent by contact inhibition, revealed a significant reduction in *PROCR* mRNA expression in quiescent cells when compared to proliferating cells (Fig. 6a). In addition, we explored *PROCR* expression changes throughout the in vitro expansion of ECFCs. We found that *PROCR* mRNA expression remained stable from P9 to P26 (Supplementary Fig. 15). Nevertheless, we observed that the frequency of ECFCs with the lowest expression of *PROCR* (bottom quartile) increased from 7% at P9, to 13% at P19, and 30% at P26. To corroborate the quiescence model findings at the protein level, we performed flow cytometry for EPCR expression. DyeCycle histograms confirmed the quiescent and proliferating phenotypes in ECFCs by cell cycle phase (Fig. 6b). Cell cycle analysis demonstrated that quiescent ECFCs show loss of G2/M peak, which was associated with a significant loss of EPCR expression, as demonstrated by the significant reduction in median fluorescence intensity (MFI) and % expression (Fig. 6b). To further confirm the relationship between EPCR expression and proliferation, we used a third model by FACS (fluorescently activated cell sorting), based on EPCR expression levels (Fig. 6c) into EPCR^low^, EPCR^mid^, and EPCR^high^ expressing ECFCs. Sorted populations were cultured and colony formation evaluated (Fig. 6d). EPCR^low^ expressing ECFCs produce significantly less colonies than EPCR^mid^ and EPCR^high^ expressing ECFCs (Fig. 6e). Furthermore, EPCR^high^ expressing ECFCs yield significantly more HPP (high proliferative potential) colonies and significantly less LPP (low proliferative potential) colonies than both EPCR^low^ and EPCR^mid^ ECFCs. In addition, co-staining of EPCR sorted cells with cell cycle analysis further demonstrates the association of EPCR expression with cell cycle status by a stepwise reduction in G1/G2M ratio from EPCR^low^ through to EPCR^high^ ECFCs (Fig. 6f, Supplementary Fig. 16). ECFCs with higher expression of EPCR exhibited the higher frequency of cells in G2M phase. These results using non-genetically modified ECFCs confirmed that EPCR expression is tightly modulated during the quiescence-proliferation dynamics in ECFCs**.**

## Discussion

High expression of EPCR has been associated with hematopoietic stem cells (HSCs) [19, 20]. In particular, EPCR-expressing HSCs show enhanced engraftment in murine bone marrow transplant models [21]. EPCR has also been used to identify vascular endothelial stem cells in murine tissues [9]. In the current study we show that EPCR is a robust cell surface marker for the identification of human cord-blood derived ECFCs. Indeed, we show a high expression of > 95% for EPCR in ECFCs from all the donors analyzed. While inter-donor variability in the percentage of EPCR-positive cells was minimal, we observed differences in EPCR expression levels, which may reflect variations in the proliferative status of the cells at the time of analysis. EPCR has been reported to be central for cell proliferation and functionality for human cord blood-derived HSCs [22] and epidermal stem cells [23] and, for the first time, we show the importance of EPCR for the proliferative capacity and functionality of ECFCs. siRNA knockdown of the gene *PROCR* coding for EPCR resulted in a significant decline of angiogenic activity, as shown by cell proliferation, migratory capacity and tube formation assays. Furthermore, sorting of EPCR^high^ expressing ECFCs exhibited the highest proliferative potential, suggesting that the level of EPCR expression as central in controlling ECFC functionality. This agrees with a study of EPCR deletion in murine endothelial cells which shows reduced sprouting angiogenesis of isolated endothelial cells in vitro and reduced post-ischemic reperfusion in vivo [13]. This study also described EPCR-dependent biased PAR-1 signaling to be crucial for regenerative angiogenesis in ischemic injury.

Interestingly, a recent study linked PAR-1 expression to ECFC function. It was shown that siRNA knockdown of PAR-1 in ECFCs led to a significant increase in proliferation in vitro, and post-ischemic revascularization in vivo [24]. Coupled with our data, this provides a thought-provoking insight into the function of EPCR-PAR-1 complex, suggesting a balance between these two receptors dictates ECFC proliferative capacity. Several studies have shown that altering EPCR expression does not affect PAR-1 expression [13, 25]. In our hands, silencing of EPCR appears to modulate PAR-1 expression in 2 out of 4 biological replicates (Supplementary Fig. 13) but overall not significant; therefore, further research is necessary to better understand the interplay between these two receptors and ECFC function.

It is well accepted that EPCR signaling in endothelial cells facilitates the activation of protein C (PC) to prevent excess levels of thrombin and promote anti-coagulation in maintenance of a homeostatic vascular system [26]. This is essential for development, evident in both mice homozygous knockout [27] and human EPCR mutation [28], proving embryonic lethal. Furthermore, the concurrent expression of EPCR and thrombomodulin within endothelial progenitors confers both anticoagulant and antifibrinolytic functions [29]. Notably, aberrant regulation of EPCR in ECFCs has been implicated in enhanced thrombin generation associated with idiopathic pulmonary fibrosis [30]. Secondly, EPCR mediates cytoprotective signaling, via its predominant ligand APC [31], to cleave and induce activation of PAR-1 and subsequent activation of downstream survival pathways including PI3K/Akt. These signaling events lead to cell survival through anti-apoptotic activity, stabilization of endothelial barrier, and anti-inflammatory response through inhibition of NFκB signaling [32]. Our *PROCR* knockout ECFCs showed a significantly impaired endothelial barrier (Fig. 2E), beyond this they exhibit a larger disruption to barrier integrity and delayed recovery after thrombin treatment. In brain endothelial cells, a PMM2 (Phosphomannomutase 2) knockdown leads to a down regulation of *PROCR* and subsequently a lower surface expression of EPCR. In this model of coagulopathy, EPCR^low^ endothelial cells demonstrate impaired integrity of endothelial cell monolayer, which agrees with our findings. This was mediated by scattering of actin filaments and altered VE-Cadherin junctions [25]. This change in barrier integrity with EPCR absence could be explained by studies in HEK293 cells (which do not express EPCR). In the absence of EPCR, APC switches to canonical cleavage of PAR-1 (at Arg1) to initiate barrier disruptive signaling responses, like thrombin, and fails to cleave Arg46 [12].

Beyond its role in coagulation and cytoprotection, there is also evidence to suggest that EPCR has a role in immune activation because it can bind directly to γδ T-cells [33]. This is due to structural similarities between EPCR and CD1/MHC-class 1. Although more research is needed to confirm activation/expansion of T-cells through this binding complex, it highlights the need for further research and raises questions about the specific role of EPCR-expressing endothelial cells in immunity. Indeed, a recent study of EPCR structure identified a novel conformation that is incompatible with PC/APC binding and may dictate EPCR properties that are still unknown [34].

Here we show that the level of EPCR expression of ECFCs dictates their proliferative potential, where *PROCR* silenced ECFCs have reduced expression of Ki67 and PCNA. Transcriptomics analysis of *PROCR* silenced ECFCs highlights a significant negative enrichment score of REACTOME CELL CYCLE pathway, and we confidently confirm that EPCR expression is responsible for progression from G1 phase of the cell cycle by reduced EdU incorporation, loss of G2M peak, and significant reduction of G1 checkpoint genes in *PROCR* silenced ECFCs. Similar results have suggested a role for Procr in controlling the proliferation of mammary stem cells, through HSP90AA1, Src, and IGFR1 [35]. In addition, we corroborated that EPCR expression dictates ECFCs proliferative status by assessing the transcriptomics at the single cell level in proliferating vs quiescent ECFCs, showing that ECFCs in a quiescent monolayer have a lower expression of *PROCR* and a significantly lower expression of EPCR on their cell surface. No previous studies have linked EPCR with contact inhibition in normal endothelial cells, however our results provide convincing evidence that EPCR expression decreased under quiescence, and this is likely associated with changes in ERKs phosphorylation and p21, as found in *PROCR* knock down ECFCs. On the other hand, *PROCR* knockdown in ECFCs led to a positive enrichment of TGFβ signaling, confirmed by an increase in TGFβ2 release and concomitant phosphorylation of SMAD2/3, suggesting that TGFβ2 acts via Alk5 which is known to produce antiangiogenic effects associated with a decrease in endothelial cell proliferation and migration [36]. It is not clear how EPCR deficiency leads to activation of TGFβ2/SMAD2/3 signaling in ECFCs, however, a relationship between EPCR and TGFβ has been established previously in human proximal tubule epithelial cells (HK-2 cells). This study proposed that EPCR occupancy by protein C switches the signaling specificity of thrombin through PAR-1 receptors leading to the inhibition of TGFβ-mediated processes such as expression of extracellular matrix proteins related to profibrotic pathology in diabetic nephropathy [37]. We observed increased phosphorylation of ERK1/2 in *PROCR* knockdown cells, a change that may also be attributed to increased TGFβ. It has been described that TGFβ can activate ERK MAP kinase via both, SMAD and non-SMAD pathways [38]. The role of ERK in regulation of epithelial to mesenchymal transition has been well established [39, 40] and ERK has also been shown to be critical in maintaining the integrity of quiescent endothelium [41].

Our findings underscore the critical role of EPCR expression in defining the identity and angiogenic capacity of ECFCs, which has significant implications for the development of ECFC-based cytotherapeutic strategies for vascular repair. Furthermore, given the application of ECFCs as ex vivo personalized disease models, such as for the factor V Leiden mutation [42], our results further establish EPCR as a key regulator of ECFC function. Additional investigations into the EPCR pathway in patient-derived ECFCs are warranted to enhance our mechanistic understanding of vascular disease pathobiology.

In conclusion, our findings establish EPCR expression as a defining marker of ECFC identity, supporting its inclusion in the immunophenotypic panel for ECFC characterization. Beyond serving as a biomarker, EPCR plays a pivotal role in modulating the angiogenic and vasoreparative function of ECFCs, primarily through the regulation of cell cycle progression.

## Methods

### ECFC isolation and culture

Endothelial colony forming cells (ECFCs) were isolated from umbilical cord blood of full-term pregnancies as previously described. Cord-blood mononuclear cells were extracted by density gradient centrifugation and plated on collagen-coated plates (Corning) in EGM-2 medium (Lonza) supplemented with 10% fetal bovine serum (FBS). Plates were cultured in 37 °C incubator with 5% CO_2_. ECFC colonies appeared after 2–3 weeks in culture with cobblestone morphology and immunophenotype confirmed by flow cytometry. ECFCs were used in experiments at passage 7–14. Methods for 3D vascular network formation within microfluidic devices, and the MSC culture aggregate formation model are described in detail in supplementary material.

### Immunofluorescence

Cells were plated onto collagen-coated cover slips in a 24-well plate at a density of 2 × 10^4^ cells/well. Cells were fixed in 4% paraformaldehyde (PFA) for 10 min at room temperature, permeabilized with 0.2% Triton X-100 in PBS for 5 min at room temperature and blocked with 5% goat serum in PBS for 1 h at room temperature. To assess distribution of EPCR staining on ECFCs, cells were stained with PROCR mouse monoclonal antibody (Proteintech) overnight at 4 °C. After washing, cells were stained with a goat anti-mouse secondary antibody Alexa Fluor 488 for 1 h at room temperature. To assess cell proliferation, cells were stained with CoraLite 488-conjugated KI-67 Polyclonal antibody (Proteintech) overnight at 4 °C. After washing, stained coverslips were mounted in Vectashield anti-fade medium with DAPI (Vector Labs) and imaged using a confocal microscope (Nikon).

### Flow cytometry

ECFCs were harvested by trypsinisation, washed, and stained in flow cytometry staining buffer (eBioscience) at a density of 5 × 10^5^ cells/100 µl test with antibodies for PROCR-FITC (Miltenyi Biotec), CD157-PE (eBioscience), CD34-APC (eBioscience). After washing in flow cytometry staining buffer, cells were resuspended in 500 µl of the same buffer and a minimum of 20,000 events recorded for each sample using the Attune NxT Flow Cytometer (Thermo Fisher Scientific). An unstained control was used to set gating strategy. Data analysis was performed using FlowJo software to assess % positive expression and median fluorescence intensity (MFI). For cell cycle analysis, the Click-iT Plus Edu Pacific Blue Flow Cytometry Assay kit (Thermo Fisher Scientific) was used, according to the manufacturer’s instructions, to assess the proportion of cells in the S phase. Cells in monolayer were treated with 10 µM Edu for 2 h, after which they are harvested, fixed, permeabilized, and stained with a reaction cocktail containing Pacific Blue fluorescent dye. Following a final wash step, cells were assessed on the Attune NxT Flow Cytometer. The event rate was kept below 10,000 events per second for all samples. To assess proportion of cells in G0/G1 and G2/M phase of cell cycle, Violet DyeCycle (ThermoFisher Scientific) was used. 1 × 10^6^ cells/ml EGM-2 medium were stained with Violet DyeCycle and incubated at 37 °C for 30 min before running on the Attune NxT Flow Cytometer (Thermo Fisher Scientific) and Attune CytPix Flow Cytometer (Thermo Fisher Scientific). Viability was assessed with 7AAD. Expression level in % was determined against untreated negative controls using FlowJo software.

### Cell transfection

Transfection was performed at 70% confluency using Lipofectamine RNAiMAX in EGM-2 (supplemented with 10% FBS), without antibiotics. Two silencer select siRNAs (Cat: 4,427,038) for PROCR were tested (ID: s20681, s20682; Thermo Fisher Scientific). S20681 had the highest efficiency and was used for all following experiments at 10 nM, for 16 h. An untransfected (Lipofectamine only) and Silencer Select negative control #1 siRNA (Cat: 4,390,843; Thermo Fisher Scientific) were included as controls. Cells were passaged and used for experiments after 48 h.

### Protein extraction and western blot

ECFCs were lysed using 1X radioimmunoprecipitation assay (RIPA) buffer containing protease and phosphatase inhibitors with EDTA (Thermo Fisher Scientific). 15 µg of protein was loaded onto 10% SDS polyacrylamide gel. Following electrophoresis, proteins were transferred onto a polyvinylidene fluoride (PVDF) membrane, and then blocked for 1 h in clear milk (Thermo Fisher Scientific). Membranes were then incubated with primary antibodies overnight at 4 °C. Antibodies used are detailed in Supplementary Table 1. After washing with TBS-T, respective horseradish-perioxidase (HRP)-conjugated secondary antibodies (Bio-Rad) were incubated for 1 h at room temperature. Chemiluminescent HRP substrate (Bio-Rad) was used to develop the blots in the G:Box F3 (Syngene). Densitometry analysis was used to quantify blots using FIJI software. ELISA methodology for TGFβ2 is described in supplementary information.

### In vitro clonogenic assay

ECFCs were seeded onto 6-well plates at a density of 200 cells per well. Medium was changed every 2–3 days and colonies were established after 8–10 days. Colonies were washed in PBS and stained using glutaraldehyde 6.0% (vol/vol) and crystal violet 0.5% (wt/vol) in distilled water for 30 min at room temperature. Plates were washed by submersion in distilled water and allowed to dry at room temperature. Wells were imaged using the EVOS Cell Imaging System (Thermo Fisher Scientific). FIJI software was used to crop, mask, and threshold the images to quantify % colony area.

### In vitro tube formation assay

Matrigel (BD Biosciences) was spiked with 10X PBS and mixed with an ECFC cell suspension in EGM-2 in a 60:40 ratio. 10 µl blobs of Matrigel were plated in a 96-well plate such that each blob contained 1.5 × 10^4^ cells. After polymerization, spots of Matrigel were covered with EGM-2 medium. After 24 h, tubes were stained with Calcein AM (Thermo Fisher Scientific) and imaged using the EVOS Cell Imaging System (Thermo Fisher Scientific). FIJI was used to crop, mask, and threshold the images to quantify % tube area.

### In vitro scratch migration assay

ECFCs were plated onto collagen coated 24-well plates at 5 × 10^4^ cells/well and cultured until they formed a confluent monolayer. A “scratch” was created in the monolayer using a 200 µl pipette tip. Phase contrast (Nikon) images of the scratch were taken and recorded as 0 h. Plates were placed in 37 °C incubator with 5% CO_2_ and cells were allowed to migrate for 6 h, when a second set of images were taken. The distance migrated was calculated as the difference in the scratch area between the 0 h and 6 h time points using FIJI.

### Barrier formation

The xCELLigence Real-Time Cell Analysis system (Agilent) was used to measure cell proliferation rate, and cell-substrate attachment in real time. Cellular impedance of electron flow by adherent cells is recorded as Cell Index. ECFCs were seeded onto E-Plates (with gold microelectrodes fused to the bottom surface) and an increasing Cell Index recorded as cells form an adherent barrier for up to 18 h. Following stable barrier formation, cell barrier was disrupted by treatment with Thrombin (0.1U/ml). Cell Index was recorded for a further 4 h to observe re-formation of cell barrier.

### Real-time qRT-qPCR

Total RNA was isolated using the Maxwell RSC simplyRNA Cells Kit (Promega) and a Maxwell RSC instrument (Promega) following the manufacturer’s instructions. RNA quantity and purity were assessed using the Nanodrop (Thermo Fisher Scientific); quality was assessed using the Bioanalyser (Agilent Technologies). Complementary DNA (cDNA) was synthesized from 1 µg of RNA using a High-Capacity RNA-to-cDNA Kit (Thermo Fisher Scientific). Real time PCR was performed using the Maxima SYBR Green qPCR mastermix (Thermo Fisher Scientific) in 10 µl reactions containing 2 µl of 1:10 cDNA dilution and 0.5 µM of gene-specific primers for 45 cycles in a LightCycler 480 (Roche). Primer sequences used are detailed in Supplementary Table 2.

### RNA sequencing (RNA-seq), analysis, and data availability

Total RNA was extracted, and quality assessed as for RT-qPCR. Libraries were prepared using KAPA RNA HyperPrep Kit with Poly A enrichment (Roche). Samples were sequenced on a NextSeq 2000 instrument (Illumina) to yield at least 40 million reads per sample, read length 2 × 50 bp PE. Reads were trimmed using Trimmomatic, v.0.32 to remove adapters and to exclude low-quality reads from the analysis. The remaining reads were then aligned to the reference genome GRCh38.p12, Gencode v. 31, using STAR aligner, v.2.5.3a. FeatureCounts function from Rsubread package (v.1.16) was used to assign reads to the corresponding genes. Only genes with more than 10 reads were retained. Gene expression read counts were exported and analyzed in the R environment (v.4.2.1) to identify differentially expressed genes (DEGs), using the DESeq2 Bioconductor library (v.1.38.3) [43]. DEGs for each comparison were defined, setting a cutoff of FDR ≤ 0.05 and Absolute log2FC > 1. Log fold change (LFC) shrinkage has been performed using the lfcShrink function with ashr methods [44].

Preranked GSEA [45] was performed for the comparison Mut vs WT, on the LFC shrinked rank for all the expressed genes using fgsea package (v. 1.24.0). The gene sets included in the GSEA analyses were obtained from C2: REACTOME or C2:KEGG categories in the MSigDB database [46] and retrieved using msigdbr package (v.7.5.1). Data were deposited on GEO under the accession number GSE282963. Transcriptomics from mouse in vivo lung injury models were retrieved from publicly available datasets GSE211335 and GSE148499. We relied on the original author’s metadata and annotation for cell filtering and identification. Our reanalysis was focused on exploring *Procr* expression for correlation analysis with genes reported to characterize vascular stem cells.

### Inducing cell quiescence

For quiescence experiments, cells were induced into a quiescent-like state by plating at a high density of 1 × 10^6^ cells/T75 culture flask. Proliferating cells from the corresponding clone of ECFCs were plated at 2 × 10^5^ cells/T75. After 3 days of culture, quiescent ECFCs had media changed to low serum media (EGM-2 medium without supplemental 10% FBS), while proliferating ECFCs were passaged at 2 × 10^5^ cells/T75. Cells were cultured for a further 24 h before use.

### Cell sorting

ECFCs were harvested and stained at density of 2 × 10^6^ cells/100 µl test using PROCR-FITC (Miltenyi Biotec). An unstained control was used to set up the gating strategy. Cells were sorted using the BD FACS Melody. Sorted populations for EPCR were denoted as EPCR^Low^, EPCR^Mid^, and EPCR^High^ expression. After sorting, cells were seeded for clonogenics assay at 200 cells per well on a collagen-coated 6-well plate. Media was changed every 2–3 days. Colonies were visible after 8–10 days, stained and imaged as previously described in clonogenics assay method. FIJI software was used to analyze total number of colonies, and colonies of high proliferative potential (HPP) (> 100 cells) and low proliferative potential (LPP) (< 100 cells).

### Statistical analysis

Statistical significance was calculated using GraphPad Prism 10 software. Differences between three groups (control untransfected, control siRNA, and *PROCR* siRNA) was evaluated using a one-way analysis of variance (ANOVA) with Tukey’s multiple comparisons test. In functional assays (clonogenics, tube formation, scratch migration, and barrier formation) and western blot quantification, control siRNA and *PROCR* siRNA groups were first normalized to the control untransfected group, and then statistical significance calculated using a paired t-test or ratio paired t-test, respectively. Analysis between three groups and two variables (i.e. phases of the cell cycle) was assessed using a two-way ANOVA. Differences between two groups were assessed using a paired t-test.

## Supplementary Information

Below is the link to the electronic supplementary material.

Supplementary file1 (PDF 1425 KB)

## Data Availability

The RNA-seq data for ECFCs with PROCR-siRNA was deposited on GEO with GSE282963. The scRNA-seq datasets of mouse lungs were downloaded from GSE211335 and GSE148499. The bulk RNA-seq datasets for ECFC transcriptomes were downloaded from GSE263058 and GSE131995. Data that supports the figures presented in the manuscript will be provided upon reasonable request to the corresponding author.
