# Supplementary material for: Human endothelial colony forming cells (ECFCs) require endothelial protein C receptor (EPCR) for cell cycle progression and angiogenic activity
Source: Angiogenesis. 2025 May 23;28(3):30. doi: 10.1007/s10456-025-09982-8 (PMC12102113; doi:10.1007/s10456-025-09982-8)
Supplement: Supplementary file 1 — Supplementary file1 (PDF 1425 KB) [file 10456_2025_9982_MOESM1_ESM.pdf]

SUPPLEMENTARY INFORMATION

Human endothelial colony forming cells (ECFCs) require endothelial protein C receptor (EPCR) for cell cycle progression and angiogenic activity

Sarah E. J. Chambers[1], Jasenka Guduric-Fuchs[1], Edoardo Pedrini[1,2], Pietro M. Bertelli[1], Chutima Charoensuk[4], Elisa Peixoto[1], Varun Pathak[1], Hamza I. Alhamdan[1,3], Ruoxiao Xie[5], Anna Krasnodembskaya[1], Judith Lechner[1], Alan W. Stitt[1], Reinhold J. Medina[1,4]

[1]Wellcome-Wolfson Institute for Experimental Medicine, School of Medicine, Dentistry, and Biomedical Science, Queen's University Belfast, Belfast, UK.

[2]Center for Omics Sciences, Vita-Salute San Raffaele University, 20132 Milan, Italy.

[3]Faculty of Medicine, Ibn Sina University for Medical Sciences, Amman, Jordan.

[4]Department of Eye and Vision Science, Institute of Life Course and Medical Sciences, Faculty of Health and Life Sciences, University of Liverpool, Liverpool, UK.

[5]Department of Materials, Design and Manufacturing Engineering, School of Engineering, University of Liverpool, Liverpool, UK.

Corresponding author: Reinhold J. Medina, R.Medina-Benavente@liverpool.ac.uk

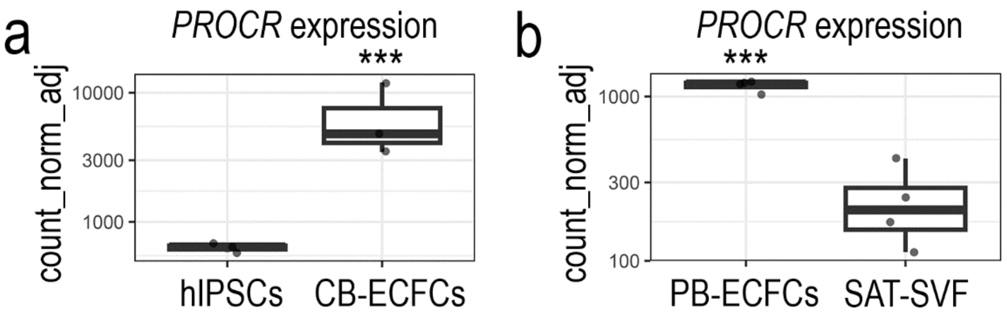

Supplementary Fig. 1. Assessment of *PROCR* mRNA expression from publicly available transcriptome datasets GSE263058 (a) and GSE131995 (b), comparing CB- and PB-ECFCs with human induced pluripotent stem cells (hIPSCs) and subcutaneous adipose tissue-derived stromal vascular fraction (SAT-SVF), respectively. ***p < 0.001.

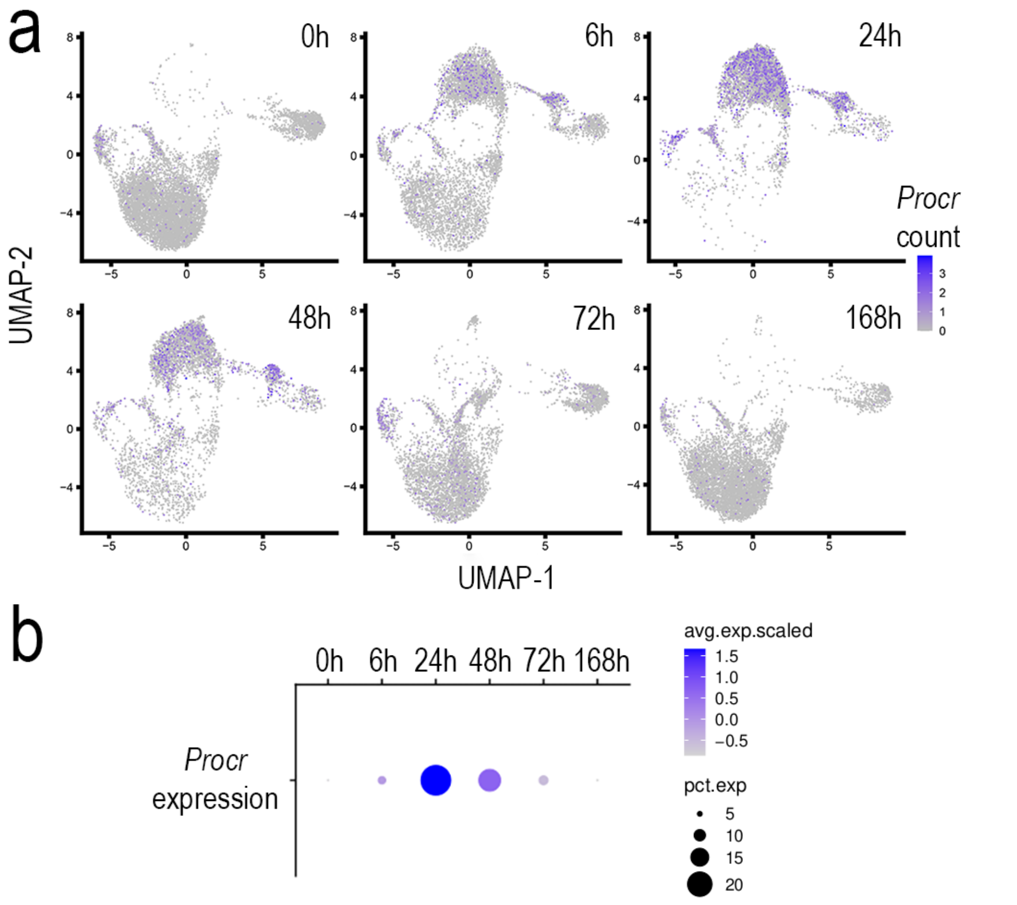

Supplementary Fig. 2. Evaluation of *Procr* mRNA expression from publicly available transcriptome dataset GSE148499 from a mouse model of LPS-induced lung injury. (a) UMAPs and *Procr* counts across time points since LPS exposure. (b) Summary metrics for *Procr* percentage expression and scaled level of expression.

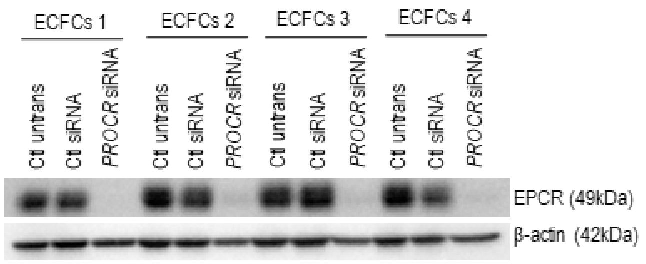

Supplementary Fig. 3. Western blot protein expression of EPCR (49kDa) in four biological replicates of ECFCs after silencing *PROCR*, compared to β-actin (42kDa), used for quantification shown in Fig.2A.

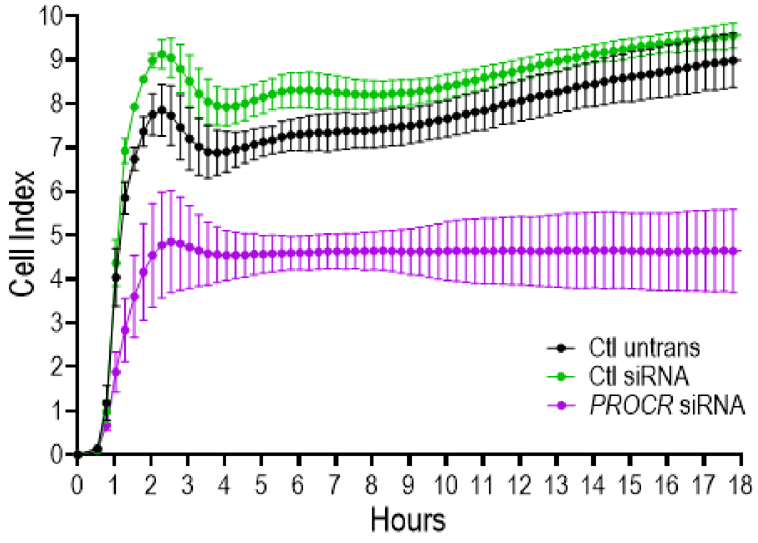

Supplementary Fig. 4. Full trace of cell index (0 to 18 hrs) from Fig. 2E showing stable barrier formation.

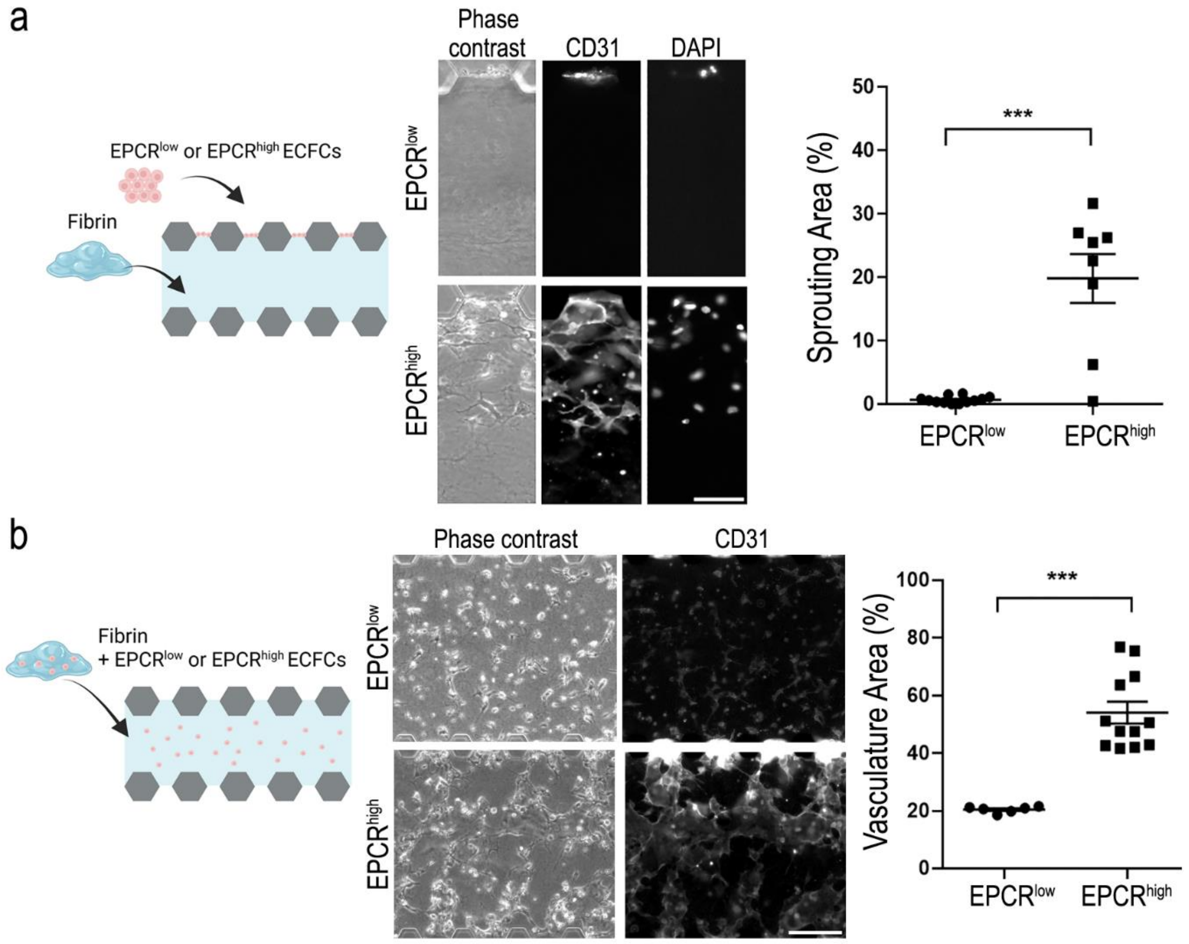

Supplementary Fig. 5. Vasculature-on-chip model with ECFCs sorted as EPCR^{low} and EPCR^{high}. Same amount of EPCR^{low} or EPCR^{high} ECFCs were loaded on microfluidic device and evaluated at 48 hours. (a) Angiogenic sprouts of EPCR^{low} at top panel and EPCR^{high} at bottom panel (left: phase contrast, middle: CD31 and right: DAPI), scale bar: 100 µm. (b) Vascular network formation of EPCR^{low} and EPCR^{high} (left: phase contrast, right: CD31), Scale bar: 200 µm. *** $p<0.001$.

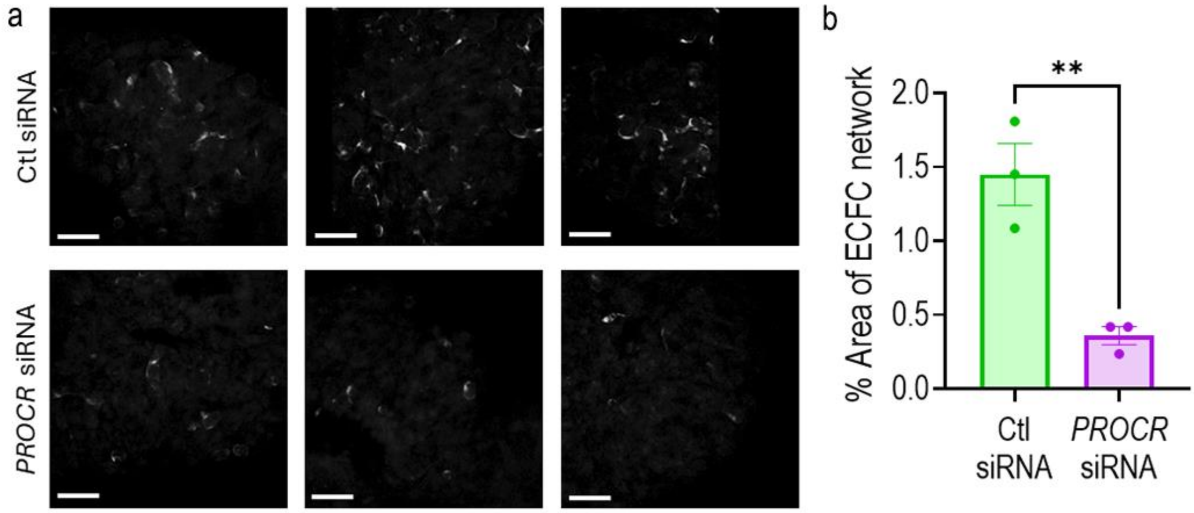

Supplementary Fig. 6. Aggregates of RFP stained ECFCs and unstained MSCs in Matrigel. RFP-ECFCs are visible in top and bottom panel, scale bar: 100 µm. Top panel shows formation of ECFC network in Control siRNA group vs. bottom panel *PROCR* siRNA ECFCs, which do not form a network within the aggregates. ** p<0.01.

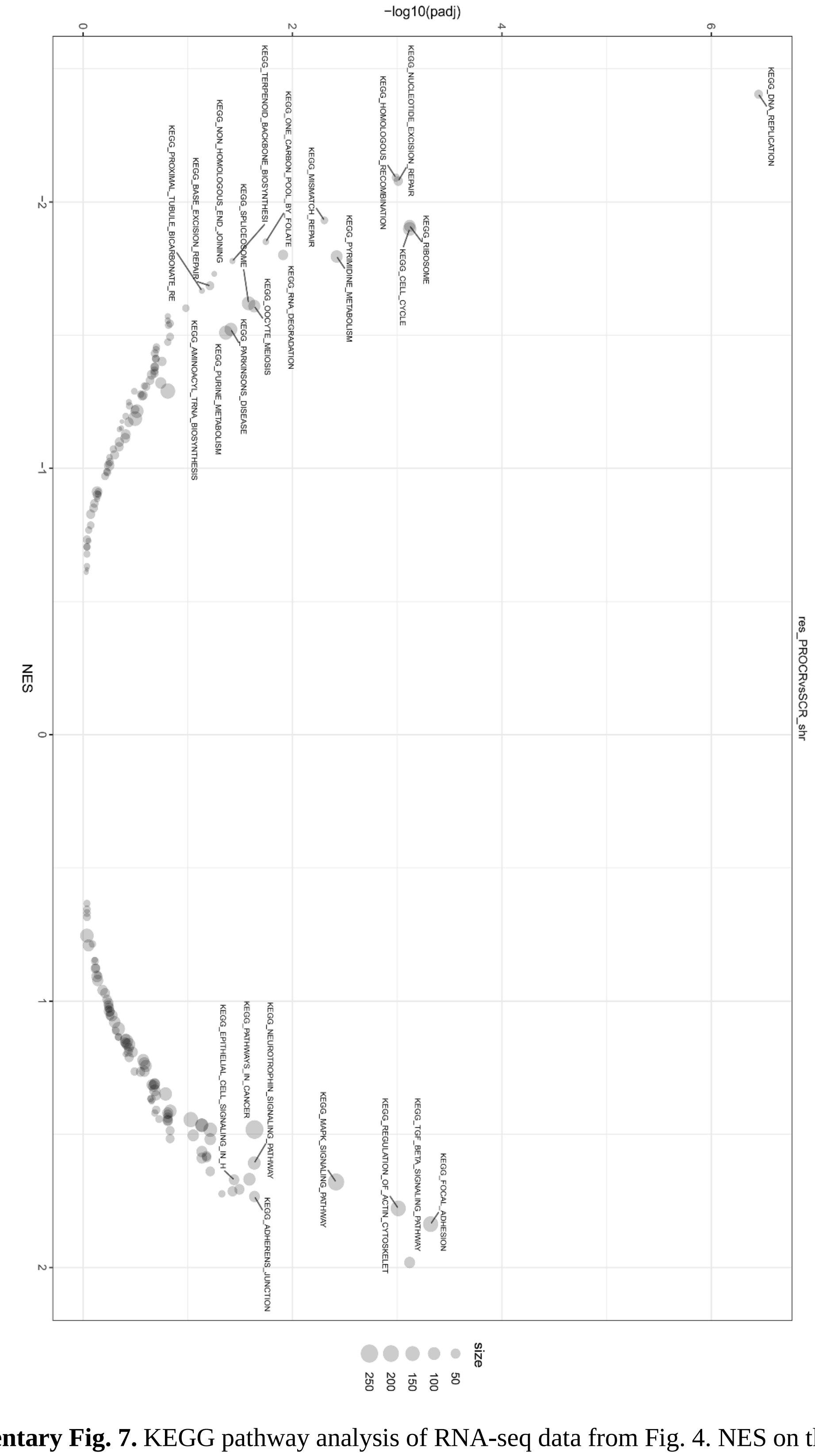

Supplementary Fig. 7. KEGG pathway analysis of RNA-seq data from Fig. 4. NES on the x-axis, and the -log10 of the adjusted p-value of the term enrichment on the y axis. Each dot represents a specific term from the KEGG annotation. The size of the dot is mapped to the size of the term. DNA_REPLICATION and CELL_CYCLE are among the top enriched terms with a negative NES. FOCAL_ADHESION and TGF_BETA_SIGNALING_PATHWAY are among the top significant enriched pathways with a positive NES.

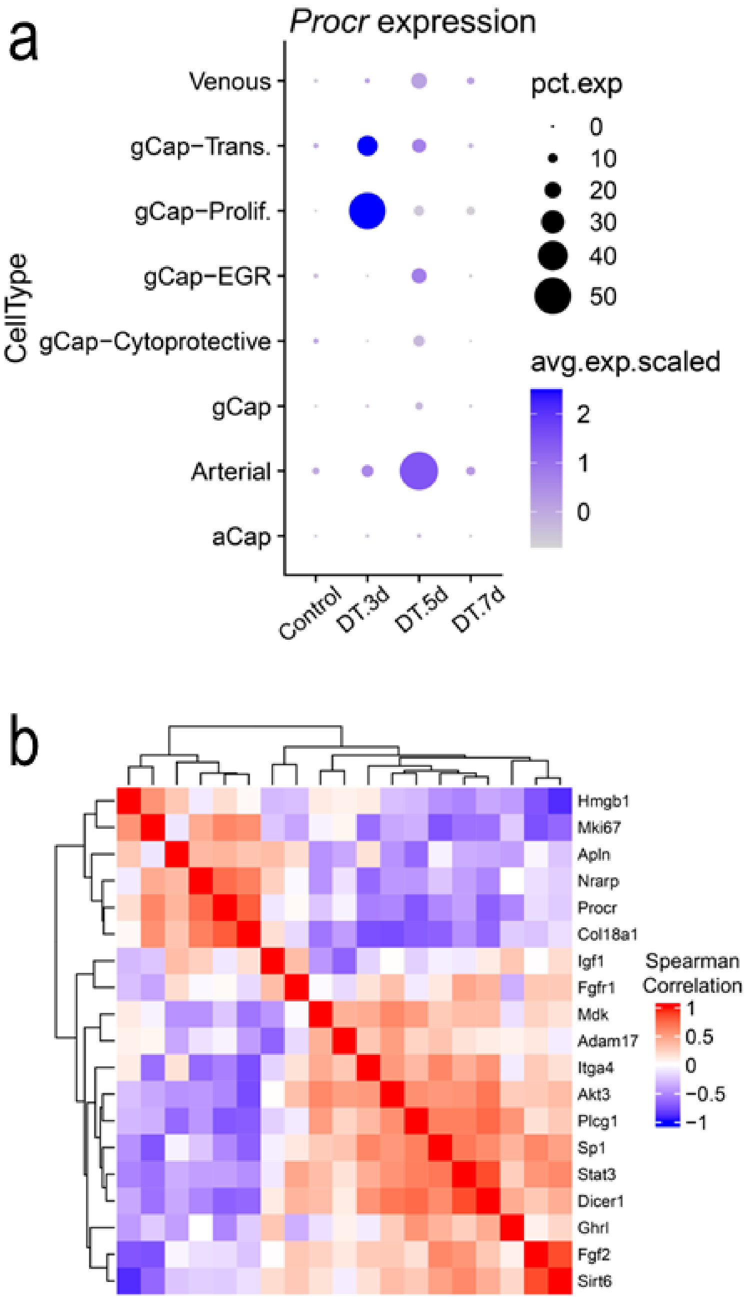

Supplementary Fig. 8. Single-cell RNA-seq dataset from a model of severe lung injury in mice (GSE211335). (a) *PROCR* expression is shown by cell type annotated by authors in the original paper. The size of the dot is mapped to the percentage of positive cells for Procr expression; the colour is mapped to the average expression per annotation. (b) Spearman correlation matrix of a panel of genes, including *Procr and* proliferation-associated genes. The expression was extracted from endothelial cells only. The average expression per gene and sample was used as unit for the correlation analysis.

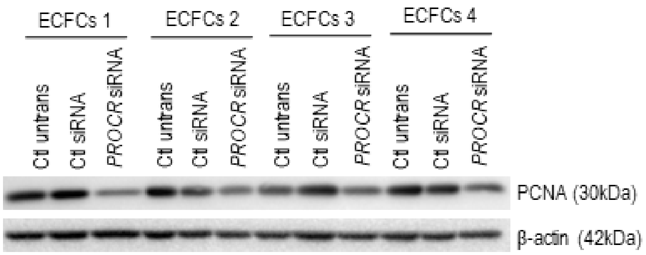

Supplementary Fig. 9. Western blot protein expression of PCNA (30kDa) in four biological replicates after silencing *PROCR*, compared to β-actin (42kDa), used for quantification in Fig. 4B.

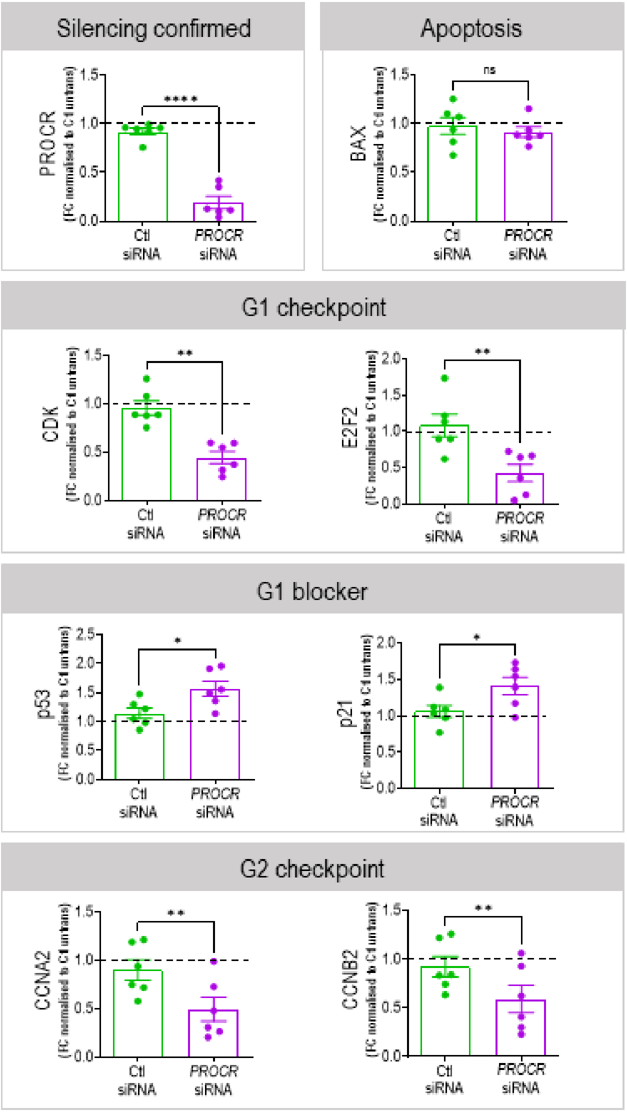

Supplementary Fig. 10. Bar graphs of RT-qPCR used to create heatmap in Fig. 4H. Six biological replicates plotted comparing Ctl siRNA vs *PROCR* siRNA (normalized to untransfected control samples) and fold change analyzed using a paired t-test, *p < 0.05, **p < 0.01; ****p < 0.0001.

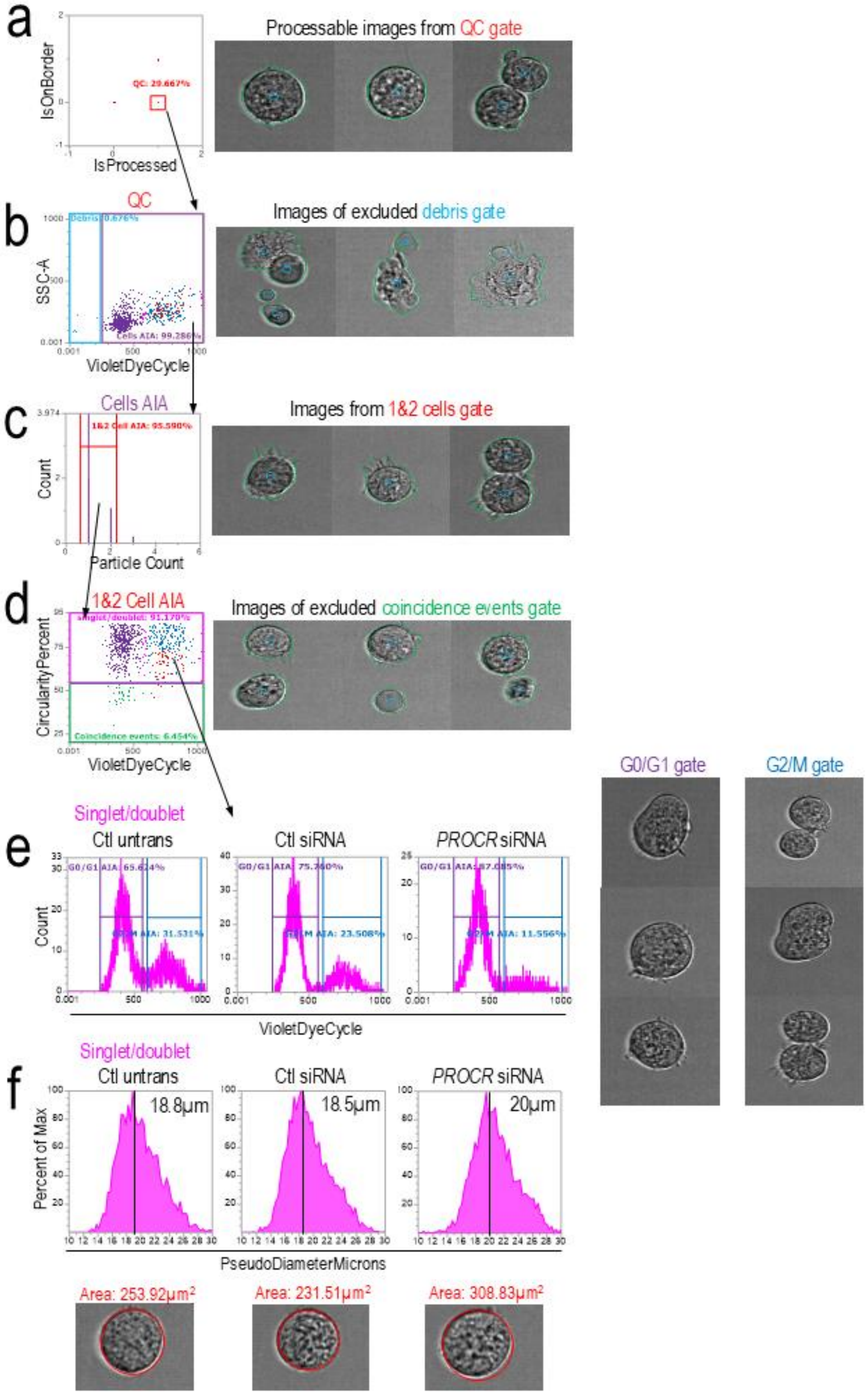

Supplementary Fig. 11. Gating strategy using automated image analysis (AIA) with the Attune CytPix Flow Cytometer to select cells which are (a) processable, (b) excluding debris, and (c) made up of 1 and 2 cells, (d) excluding coincidence events. (e) Violet dye cycle histogram and representative images of cells in G0/G1 gate and G2/M gate. (f) Histograms of pseudo-diameter (in µm) of cells in Ctl untrans, Ctl siRNA, and PROCR siRNA groups. Vertical line shows the peak value (mode) in µm. Below, are representative images of single cells from each group, with cell area calculated (red line) using image analysis within the Attune Cytometric Software.

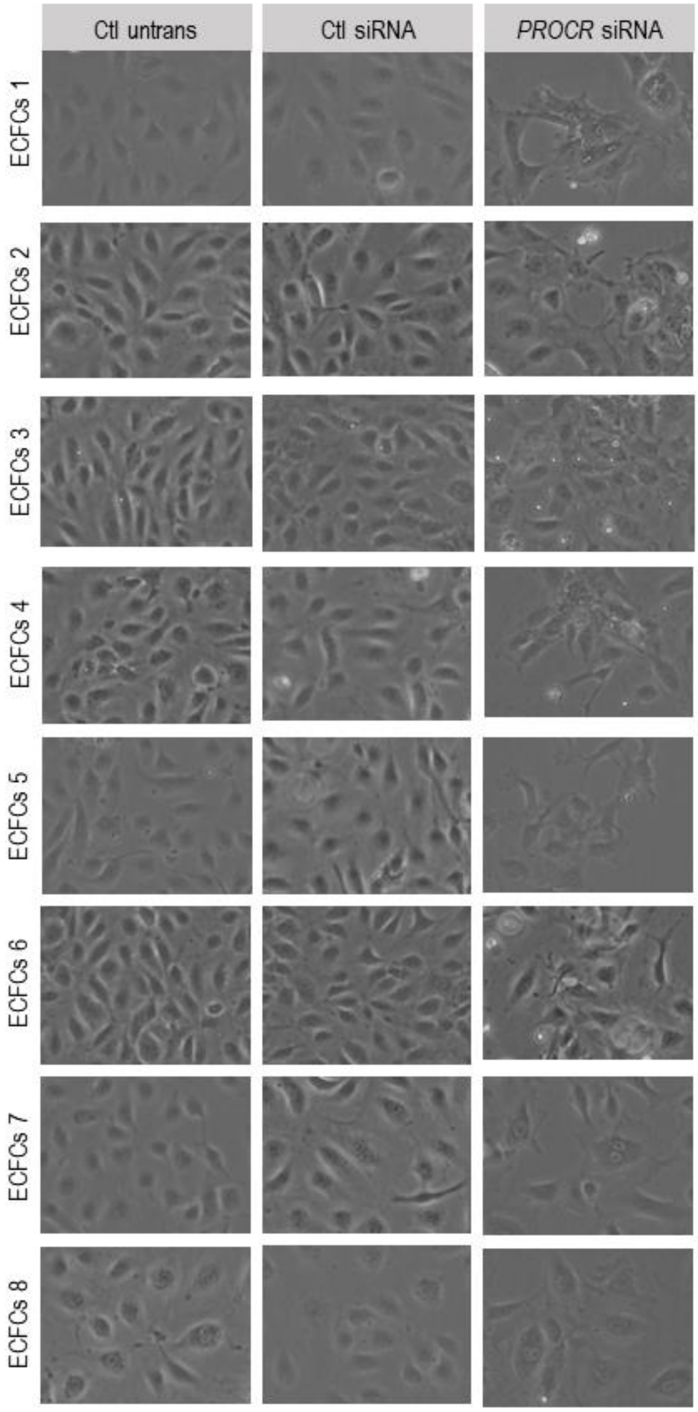

Supplementary Fig. 12. Changes in morphology observed in *PROCR* siRNA ECFCs compared to Ctl siRNA and Ctl untransfected cells in phase contrast images from eight biological replicates.

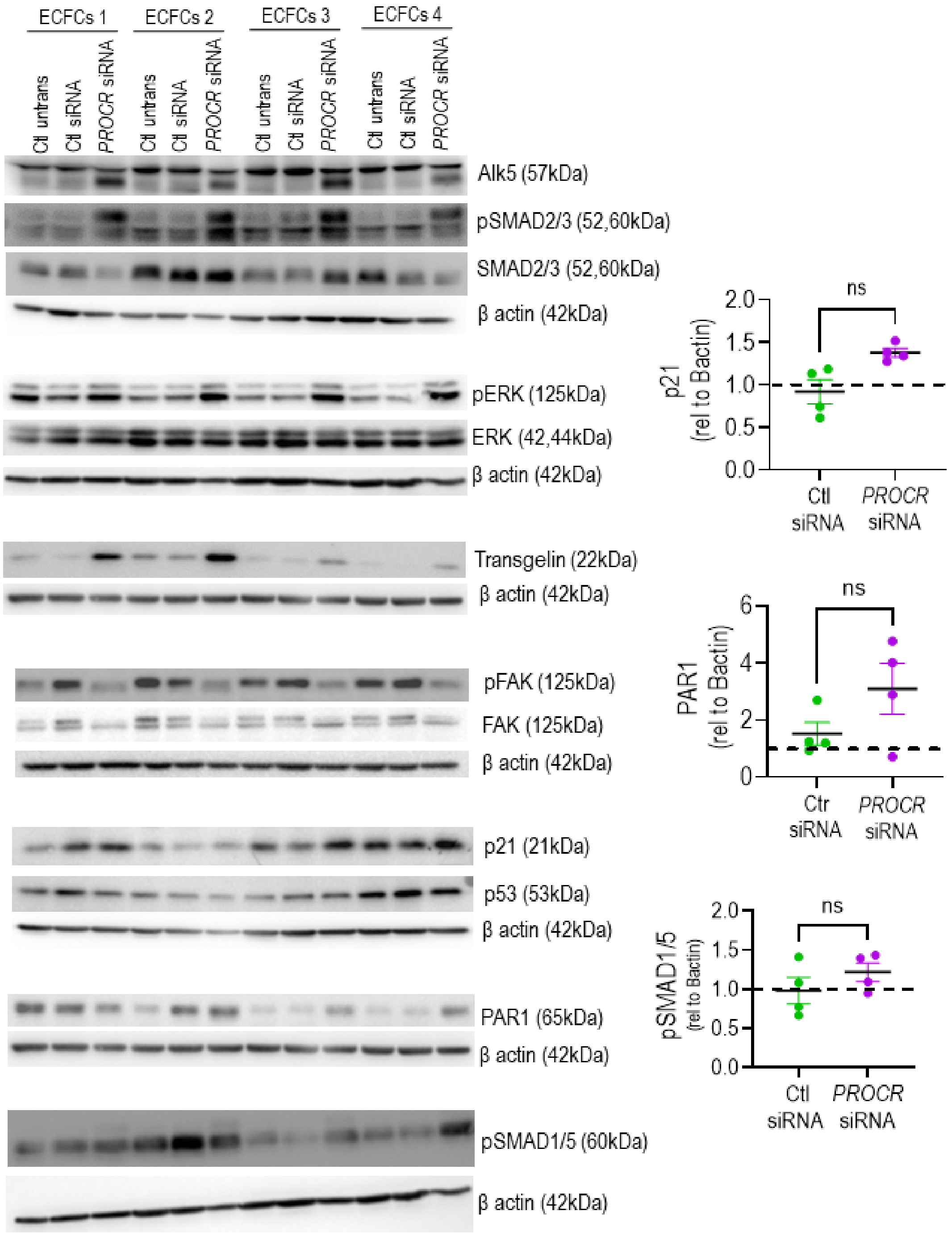

Supplementary Fig. 13. Western blot protein expression of four biological replicates probed for Alk5 (57kDa), pSMAD2/3 (52, 60kDa), SMAD2/3 (52, 60kDa), pERK (125kDa), ERK (42,44kDa), Transgelin (22kDa), pFAK (125kDa), FAK (125kDa), p21 (21kDa), p53 (53kDa), PAR1 (65kDa), and pSMAD1/5 (60kDa). Densitometry analysis is relative to β-actin (42kDa), normalized to Ctl untrans and analyzed by ratio paired t-test, ns = not significant.

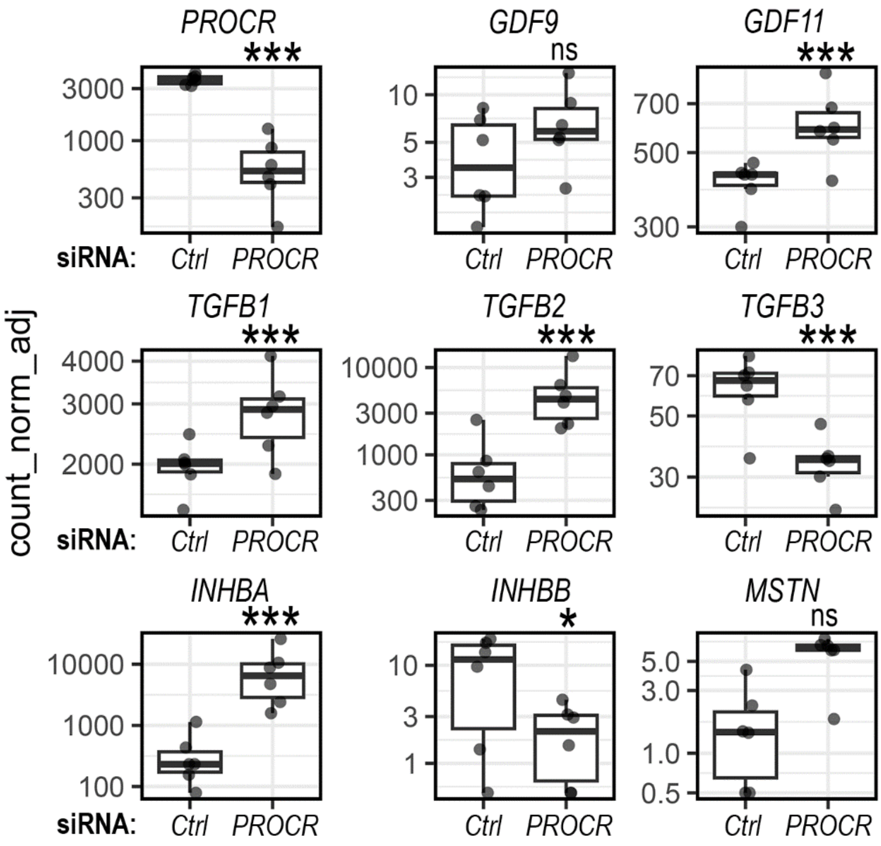

Supplementary Fig. 14. Evaluation of changes in gene expression for Alk5 ligands when *PROCR* was knocked down in ECFCs. ***p<0.001, ns= not significant.

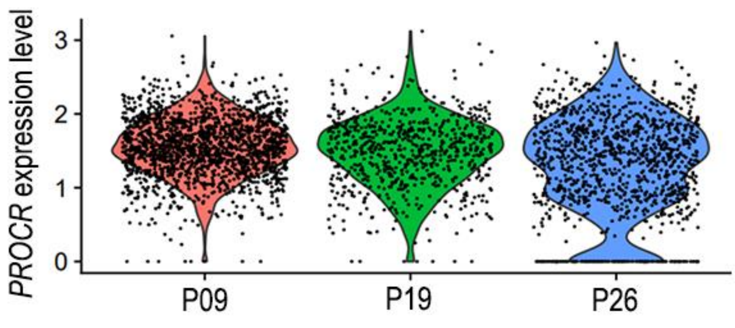

Supplementary Fig. 15. Assessment of *PROCR* expression at the single cell level, from scRNAseq dataset, throughout the in vitro lifespan of ECFCs from passage 9 (P09) to their Hayflick limit at passage 26 (P26).

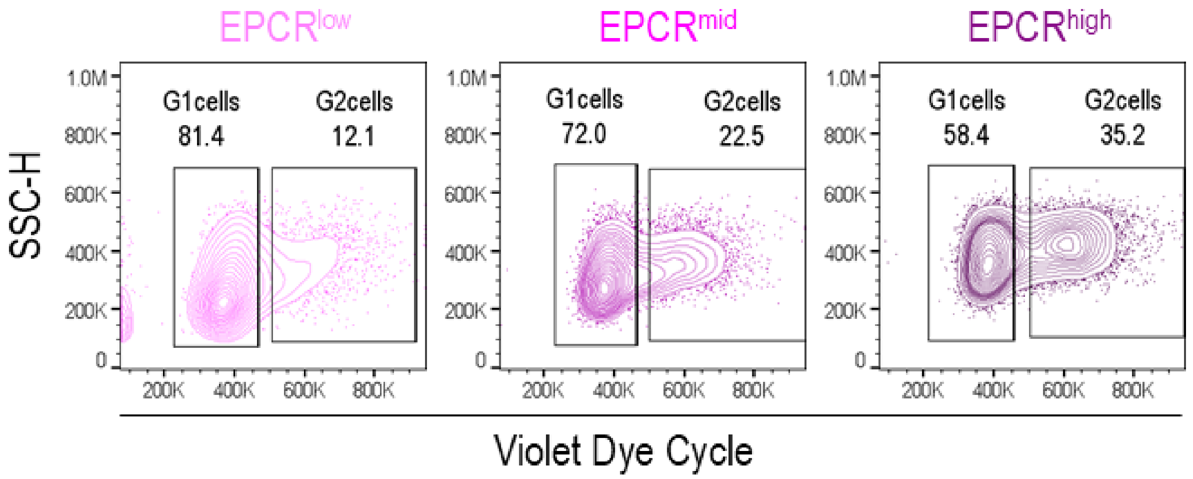

Supplementary Fig. 16. Flow cytometry analysis of Violet Dye Cycle-stained cells after gating on EPCR^{low}, EPCR^{mid}, and EPCR^{high} populations (Fig. 6C). Dot plots show gating strategy of G1 and G2M phase cells. This was used to quantify and plot G1/G2M ratio in Fig. 6F.

Supplementary Table.1 Antibody Dilutions

**Antibody**	**Company**	**Dilution**
EPCR	Protein Tech	1/1000
PCNA	Thermo Fisher Scientific	1/1000
Alk5	Cell Signalling Technology	1/1000
pSMAD2/3	Cell Signalling Technology	1/1000
pERK	Cell Signalling Technology	1/1000
p21	Abcam	1/1000
p53	Abcam	1/1000
Transgelin	Abcam	1/1000
FAK	Invitrogen	1/1000
pFAK	Invitrogen	1/1000
B-actin	Cell Signalling Technology	1/3000

Supplementary Table.2 Primer Sequences

**Gene**	**Forward Primer**	**Reverse Primer**
PROCR	GCTCAATGCCTACAACCGCAC	CGAAGTGTAGGAGCGGCTTGTT
CDK	CAGGGGATTGTGTTTTGTCA	TCTGAATCCCCATGGAAAAG
E2F2	CTGCGTTCACAGGTGTTTCT	AAGTCGAAGTTCCATCGCTC
P21	CTGCGTTCACAGGTGTTTCT	AAGTCGAAGTTCCATCGCTC
P53	TCCCTGGATTGGCAGCCAGACT	TCCATTGCTTGGGACGGCAAGG
CCNA2	GGTACTGAAGTCCGGGAACC	GCTTTCCAAGGAGGAACGGT
CCNB2	ATTGGAAGTCATGCAGCACA	TCTGACGGCAACTTCAACTG
BAX	TCTGACGGCAACTTCAACTG	GAGGAAGTCCAATGTCCAGCC
RPL30	GCTGGAGTCGATCAACTCTAGG	CCAATTTCGCTTTGCCTTGTC

Supplementary Methods

3D vascular network formation in microfluidic devices

To obtain EPCR^{low} and EPCR^{high} populations, ECFCs at passage 12 were harvested and washed in staining buffer (5% FBS in 1 x PBS). Cells were stained with PE-conjugated anti-human EPCR antibody (BioLegend) for 30 minutes at 4°C in the dark. A matching PE-conjugated isotype control antibody was used in parallel to assess non-specific binding. After staining, cells were washed twice and resuspended with staining buffer. EPCR^{low} and EPCR^{high} populations were gated and sorted using a FACSAria III cell sorter (BD Biosciences). To perform the sprouting assay, fibrin-collagen gel was prepared using 5 mg/mL fibrinogen (Sigma) and 0.4 mg/mL type I collagen (Gibco) in PBS, and kept on ice. Thrombin (0.5 U/mL, Merck) was added to initiate polymerization. The gel mixture was then loaded into channel 3 of a five-channel microfluidic device and incubated at 37°C in a humidified chamber with 5% CO_2 for 1 hour to allow polymerization. Cells were resuspended in EGM-2 medium (Lonza) at 2×10^6 cells/mL. A 20 µL cell suspension was injected into channel 2 and incubated vertically at 37°C for 30 minutes to promote cell attachment to the gel wall. EGM-2 medium was then added to channels 2 and 4. To induce the formation of vascular networks, 20 µL of EPCR^{low} or EPCR^{high} cells with concentration 1x107 cells/mL were prepared in EGM-2. Cell suspensions were mixed with the fibrin-collagen gel and thrombin, then introduced into channel 3 of the microfluidic device. The devices were incubated at 37°C in 5% CO_2 for 30 minutes. After polymerization the medium was injected into channel 2 and 4. For sprouting and vasculogenesis assays, devices were cultured for 72 hours in a humidified chamber, with daily medium changes. After 3 days, cells were fixed using 4% paraformaldehyde for 30 minutes at room temperature. Blocking was performed using 5% BSA in 1 x PBS for 3 hours at room temperature. Cells were stained with anti-CD31 antibody (R&D) overnight at 4◦C, followed by incubation with a secondary antibody (Invitrogen) for 3 hours at room temperature. Nuclei were counterstained with DAPI (Invitrogen). Imaging was performed using a Zeiss Observer Apotome microscope, and image analysis was carried out using Fiji (ImageJ).

MSC culture and aggregate formation

Human Bone Marrow-Mesenchymal Stem cells (BM-MSC) were cultured in T25 flasks and maintained in DMEM-F12 medium (Thermo Fisher Scientific) supplemented with 10% FBS and 1% Pen/Strep. BM-MSC were used in passage 5 in the aggregate formation by coculturing them with ECFCs, which maintained in EGM2 medium and used at passage 14. A 40% Matrigel solution was prepared by mixing Matrigel (Corning 356231) with EGM2 medium at 4:6 ratio. The mixture was dispensed into the wells of a 96-well plate (80 µL per well) to establish a 3D supportive base for cell mixture. The plate was then incubated at 37°C for at least 40 minutes to allow polymerization of the Matrigel matrix. Aggregates were generated for each condition of RFP- positive ECFCs (SiRNA Scrambled control, PROCR SiRNA) by co-culturing them with BM-MSCs at 7 ECFC: 2 BM-MSC ratios with a total of 9×10^4 cells per aggregate. The cells were combined at specific ratio and then resuspended in 5% Matrigel in EGM2 medium, which then dispensed onto pre-polymerized 40% Matrigel (100 µL of seeding cell suspension per well). EGM2v medium was replaced every 48 hours, and the aggregates were fixed at day 8.

ELISA measurement of TGFβ2 in cells treated with ALK5 inhibitor

Following transfection with PROCR and control SiRNA, ALK5 inhibitor SB525334 (RayBiotech) was added to the cell media at the concentration of 10 µM for 72 hours. Cells from three ECFC donors were used. Media was collected and TGFβ2 concentration was measured using Human TGF-beta 2 Quantikine ELISA Kit (R&D systems). TGFβ2 levels were assessed in duplicates following manufacturer's instructions. Optical density was determined using a microplate reader set to 450 nm. ELISA data were analysed using a four-parameter logistic (4PL) regression model to generate standard curves and calculate sample concentrations.
